# RNA-Seq based transcriptome analysis during bovine viral diarrhoea virus (BVDV) infection

**DOI:** 10.1186/s12864-019-6120-4

**Published:** 2019-10-24

**Authors:** Cun Liu, Yanhan Liu, Lin Liang, Shangjin Cui, Yanming Zhang

**Affiliations:** 1 College of veterinary medicine, Northwest A&F University, Yangling, 712100 Shaanxi China; 2 Institute of Animal Sciences, Chinese Academy of Agricultural Sciences, Beijing, 100193 China; 3 Beijing Observation Station for Veterinary Drug and Veterinary Biotechnology, Ministry of Agriculture, Beijing, 100193 China; 4 College of Veterinary Medicine, China Agricultural University, Beijing, 100193 China

**Keywords:** Bovine viral diarrhoea virus, RNA-Seq, Transcriptome analysis, Pathogen-host interactions

## Abstract

**Background:**

Bovine viral diarrhoea virus (BVDV) is the member of the genus *Pestivirus* within the *Flaviviridae* family and responsible for severe economic losses in the cattle industry. BVDV can employ ‘infect-and-persist’ strategy and ‘hit-and-run’ strategy to remain associated with hosts and thus contributes to BVDV circulation in cattle herds. BVDV have also evolved various strategies to evade the innate immunity of host. To further understand the mechanisms by which BVDV overcomes the host cell innate immune response and provide more clues for further understanding the BVDV-host interaction, in this descriptive study, we conducted a investigation of differentially expressed genes (DEGs) of the host during BVDV infection by RNA-Seq analysis.

**Results:**

Our analysis identified 1297, 1732, 3072, and 1877 DEGs in the comparison groups mock vs. MDBK cells infected with BVDV post 2 h (MBV2h), mock vs. MBV6h, mock vs. MBV12h, and mock vs. MBV24h, respectively. The reproducibility and repeatability of the results were validated by RT-qPCR. Enrichment analyses of GO annotations and KEGG pathways revealed the host DEGs that are potentially induced by BVDV infection and may participate in BVDV-host interactions. Protein-protein interaction (PPI) network analyses identified the potential interactions among the DEGs. Our findings suggested that BVDV infection induced the upregulation of genes involved in lipid metabolism. The expression of genes that have antiviral roles, including *ISG15*, *Mx1*, *OSA1Y*, were found to be downregulated and are thus potentially associated with the inhibition of host innate immune system during BVDV infection. The expression levels of *F3*, *C1R*, *KNG1*, *CLU*, *C3*, *FB*, *SERPINA5*, *SERPINE1*, *C1S*, *F2RL2*, and *C2*, which belong to the complement and coagulation signalling cascades, were downregulated during BVDV infection, which suggested that the complement system might play a crucial role during BVDV infection.

**Conclusion:**

In this descriptive study, our findings revealed the changes in the host transcriptome expression profile during BVDV infection and suggested that BVDV-infection induced altering the host’s metabolic network, the inhibition of the expression of antiviral proteins and genes within the complement system might be contributed to BVDV proliferation. The above findings provided unique insights for further studies on the mechanisms underlying BVDV-host interactions.

## Background

Bovine viral diarrhoea viruses (BVDV), with positive, non-segment, single stranded RNA genome, comprise a heterogeneous group of viruses which belong to the genus *Pestivirus*, the *Flaviviridae* family [1]. BVDV is a globally-distributed virus and its infection has been detected not only in domesticated ruminants, but also in wild ruminants and wild boars [2–7]. It is responsible for numerous clinical disease syndromes in cattle and severe economic losses in the cattle industry.

BVDV is divided into two species, namely, Pestivirus A and Pestivirus B, which are formerly known as BVDV 1 and BVDV 2, respectively [8]. Given the genetic heterogeneity of BVDV, both species are divided into multiple genotypes. To date, Pestivirus A is divided into at least 17 genotypes, while Pestivirus B is divided into 4 genotypes [9]. Both BVDV species have two biotypes, including the non-cytopathogenic biotype (NCP) and the cytopathogenic biotype (CP) [1]. NCP BVDV can establish persistent infection by crossing the placenta and infecting the foetus, which is born as a persistently infected (PI) calf after 30 to 150 days of pregnancy [10]. PI cattle are the most important sources of BVDV infection and are the key factors affecting BVDV circulation in farms. PI cattle can carry and shed viruses throughout their lifetimes, which in turn produces more PI calves from PI cattle. Therefore, the detection and elimination of PI cattle are of great significance for the prevention and control of BVD/MD in cattle herds.

BVDV replication, occurring in the cellular cytoplasm, is a multistage process. The binding and entry of BVDV begin with attachment or interaction of the virion with specific host cell receptors, followed by internalization and pH dependent fusion of the viral envelope and cell membrane [11, 12]. After entry into the host cell is complete, viral RNA is released into the host cell cytoplasm and RNA translation begins. Initiation of the translation process is mediated by the IRES which binds specifically to the 40S ribosomal subunit in the absence of any additional translation initiation factors [13]. Then, the 80S complex was formed at the start codon AUG and initiate the translation of polyprotein which are cleaved into structural and non-structural proteins [14]. Viral non-structural proteins assemble into a functional replicase complex to catalyze transcription of positive-sense RNA into full-length complementary strand negative-sense RNA which provide template for the replicase complex to synthesize additional positive-sense RNA molecules [15]. The replication process begins with a positive-strand replicase complex comprised of viral and cellular components formed at the 3′ terminus of the genome. Progression from initiation to elongation occurs after the synthesis of nascent RNAs 8–10 nucleotides in length [16]. Elongation mediated by viral-polymerase displaces the positive strand from the RI template, allowing recycling of the template while elongation of the prior nascent strand continues. Then, capsid protein binds to viral genomic RNA and identifies the cytoplasmic region containing the envelope protein that budding in the endoplasmic reticulum cavity [17–19]. BVDV virions appear to mature in intracellular vesicles at the Golgi apparatus or endoplasmic reticulum. The intact virions are released by exocytosis with detection reported as early as 8 h post infection [20].

Viruses employ two different strategies to remain associated with hosts, including ‘infect-and-persist’ strategy and ‘hit-and-run’ strategy [21]. Viruses that use the ‘infect-and-persist’ strategy rather than the ‘hit-and-run’ strategy are capable of efficiently evading the immune defence systems of the susceptible hosts and have evolved elaborate mechanisms of evasion [22, 23]. BVDV can employ both strategies to remain associated with hosts and thus contributes to BVDV circulation in cattle herds [23].

The innate immunity serves as the first line of defence against foreign microbial pathogens. Considering that full induction of antiviral innate immunity severely limits viral replication, viruses have evolved diverse strategies to evade the innate immunity [24]. Current studies have shown that BVDV can evade the innate immune system via multiple strategies, such as inhibition of IFN synthesis, inhibition of IFN protein activity, and interference with expression or activity of the IFN-induced antiviral effector proteins [24]. Degradation of IRF-3 induced by BVDV N^pro^ has been shown to decrease the production of interferon [25, 26]. BVDV inhibits interferon production and lymphocyte apoptosis through the degradation of viral RNA mediated by E^rns^ [27, 28]. The mechanisms underlying BVDV-host interactions and the pathogenesis of BVDV infection are complex. Successful evasion from the host immune defence system is the basis for persistent BVDV infection. Although BVDV employs several strategies to evade the immune system, especially the innate immune system, these strategies do not reveal the complete mechanisms underlying BVDV-host interactions during infection. The present study aimed to investigate the changes in gene expression and identify the active or suppressed signal pathways induced by BVDV during the early stage of virus infection, which could be associated with the BVDV-host interactions. This work provided a theoretical basis for further studies on BVDV-host interactions during the early stage of viral infection.

## Results

### Growth curves of BVDV with different MOIs

**Fig. 1:**
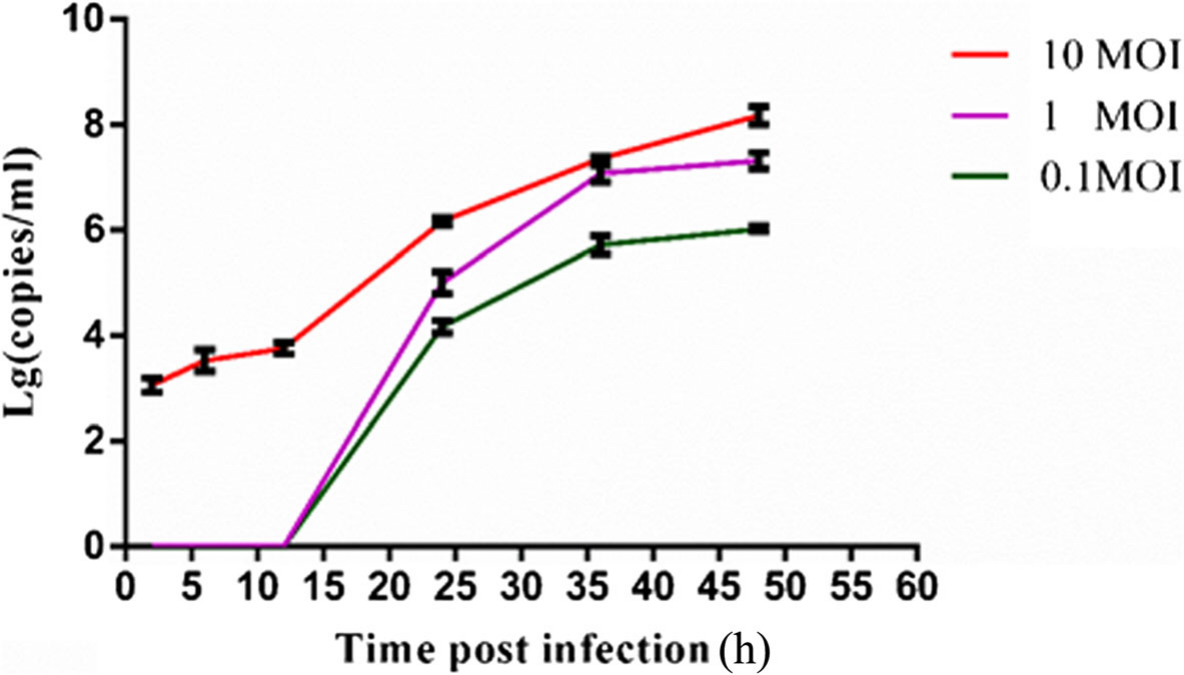
Growth curves of BVDV in MDBK cells. BVDV showed similar proliferation trend after MDBK cell were inoculated with virus of different MOI. Each data point represented the average of three replicates. The error bar indicates standard deviation

To determine the period of intracellular replication, the infection dynamics of BVDV on MDBK cells was defined using one-step growth curve method. The growth curves of BVDV at 0.1, 1, and 10 MOI were determined by measuring the changes in the number of copies of the BVDV genome in the cell supernatant by RT-qPCR (Fig. 1). The growth curves indicated that the number of viruses remained more or less stable in the cell supernatant within 12 h-post infection, which was followed by a rapid increase in the viral load in cell supernatant. This observation suggested that it is the intracellular replication period of BVDV within 12 h post-infection. After 12 h post-infection, the virus began to be released from cells in large quantities. Due to all stages of BVDV life cycle were observed within 24 h post infection, the samples used for transcriptome sequencing were collected at the 2, 6, 12, and 24 h time points.

### Evaluation of transcriptome sequencing data

At least 6.0 Gb of clean data from each sample was obtained from transcriptome sequencing from Illumina NovaSeq 6000 platform and were available for further expression level analysis after quality control. Q30 percentages of clean data for all samples were higher than 94.36%, and the GC contents of the clean data for all samples ranged between 54.3 to 55.32% (Table 1). For further analysis, the high-quality clean reads were mapped to the reference *Bos taurus* genome (UMD3.1). Approximately 95.97 to 96.32% of clean reads were successfully mapped to the reference *Bos taurus* genome (UMD3.1). In addition, 91.58-93.44% of clean reads were uniquely mapped to the reference *Bos taurus* genome (UMD3.1). A total of 53,929 transcripts were reference-based assembled from the mapped data using StringTie software. Length distribution statistics of transcripts indicated that the length of more than half of the transcripts were greater than 1800 bp (Additional file 1: Table S1). After removing low-quality and redundant sequences, a total of 53,391 transcripts were obtained, corresponding to 26,740 reference transcripts. Simultaneously, 26,975 genes were assembled from the mapped reads, out of which 24,616 genes successfully mapped to the reference *Bos taurus* genome (UMD3.1). A total of 26,651 novel transcripts and 2359 of novel genes were predicted by gffcompare software and were subsequently annotated by comparison with the NR, Swiss-Prot, and Pfam databases (Additional file 1: Table S2). Further studies were performed based on the reference genes.

**Table 1:** Summary statistics for sequence quality control and mapped data of samples

Sample	Raw reads	Raw bases	Clean reads	Clean bases	Error rate(%)	Q30(%)	GC content(%)	Total mapped	Multiple mapped	Uniquely mapped
Mock1	59,871,314	9.04E+ 09	58,364,222	8.64E+ 09	0.0127	94.47	54.69	56,091,717	2,022,528	54,069,189
Mock2	62,652,690	9.46E+ 09	61,106,134	9.05E+ 09	0.0127	94.46	54.84	58,825,929	1,973,290	56,852,639
Mock3	62,037,832	9.37E+ 09	60,491,658	8.96E+ 09	0.0127	94.46	54.75	58,228,017	1,853,339	56,374,678
MBV2h1	59,861,152	9.04E+ 09	59,295,566	8.86E+ 09	0.0232	95.94	54.73	57,077,611	2,775,919	54,301,692
MBV2h2	57,685,384	8.71E+ 09	57,103,346	8.53E+ 09	0.0231	96.11	54.82	54,969,915	2,673,949	52,295,966
MBV2h3	55,726,700	8.41E+ 09	55,147,954	8.24E+ 09	0.0231	96.02	55	53,040,252	2,267,352	50,772,900
MBV6h1	61,244,856	9.25E+ 09	60,686,854	9.06E+ 09	0.0227	96.53	55.32	58,408,773	2,607,450	55,801,323
MBV6h2	56,301,976	8.5E+ 09	55,767,750	8.34E+ 09	0.023	96.22	54.82	53,609,101	2,131,215	51,477,886
MBV6h3	61,993,652	9.36E+ 09	61,351,120	9.16E+ 09	0.0234	95.78	54.81	58,953,749	2,347,841	56,605,908
MBV12h1	55,707,520	8.41E+ 09	55,210,364	8.25E+ 09	0.023	96.23	54.48	53,122,918	2,080,378	51,042,540
MBV12h2	55,239,962	8.34E+ 09	54,712,404	8.18E+ 09	0.023	96.18	54.84	52,508,770	2,092,552	50,416,218
MBV12h3	57,751,894	8.72E+ 09	57,242,562	8.56E+ 09	0.0228	96.39	54.71	54,983,532	3,032,684	51,950,848
MBV24h1	61,825,850	9.34E+ 09	60,299,100	8.93E+ 09	0.0127	94.49	54.58	58,047,262	2,112,347	55,934,915
MBV24h2	59,229,538	8.94E+ 09	57,746,066	8.56E+ 09	0.0128	94.36	54.43	55,605,127	1,780,562	53,824,565
MBV24h3	58,448,052	8.83E+ 09	57,040,466	8.45E+ 09	0.0126	94.56	54.3	54,944,177	1,642,785	53,301,392

### Functional annotation and classification

To generally described the functions and pathways of genes obtained from RNA-Seq, functional annotation and classification were performed by comparing the sequences with the GO, COG, and KEGG databases. These analyses could provide general information for function and classification of genes or transcripts, which can only annotated up to level 2. GO is a gene function classification system that is often used to describe gene properties. Annotation results for the three databases were shown in Additional file 2: Figure S1. In the present study, GO terms were extracted using Blast2GO software. Genes annotated by GO database were classified into the three GO categories, namely, biological progress (BP), cellular component (CC), and molecular function (MF). The genes were assigned to a total of 63 GO terms, which were predominantly comprised of cellular process, single-organism process, biological regulation, regulation of biological process, metabolic process, cell, cell part, organelle, binding, catalytic activity, signal transducer activity. The assignments of the top 20 level 2 GO terms are shown in Additional file 2: Figure S2. Genes annotated with the COG database were best matched to four gene functional types (information storage and processing, cellular processes and signalling, metabolism, poorly characterized) and assigned to 24 functional categories. The top three functional terms were post-translational modification, protein turnover, chaperones, translation, ribosomal structure and biogenesis, and signal transduction mechanisms (Additional file 2: Figure S3).

The KEGG database contains abundant pathway information that can help understand the biological functions of genes at the systems level. In the present study, gene annotations using KEGG pathway database was performed using KOBAS (Additional file 2: Figure S4). Based on KEGG analysis, the genes were grouped into six first categories, corresponding to 43 s categories. The top five second-category KEGG pathways included signal transduction, sensory system, cancers overview, immune system, endocrine system, and transport and catabolism.

### Differential gene expression analysis

**Fig. 2:**
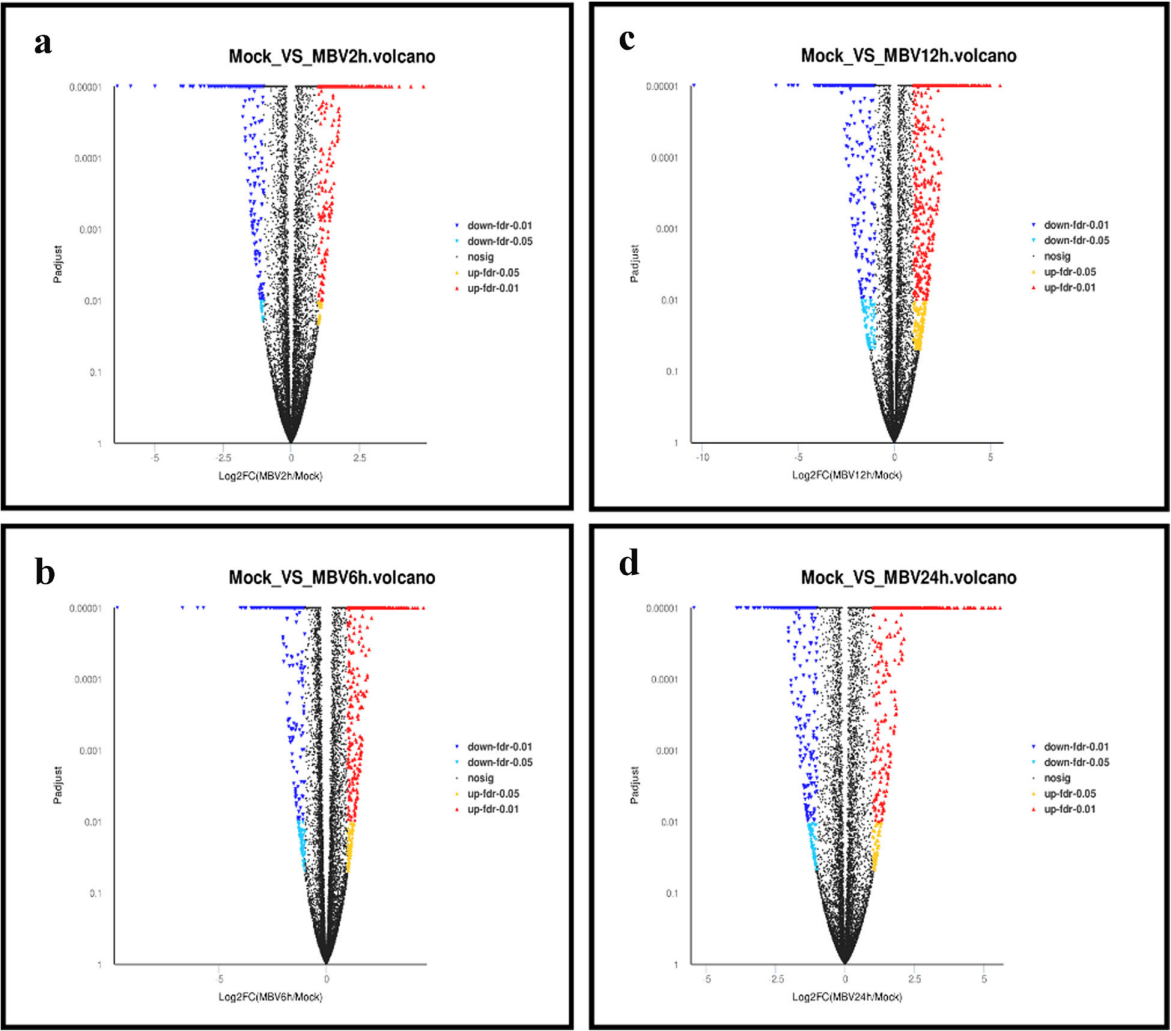
Volcano plot of global DEGs in different comparison groups, Mock VS MBV2h (**a**), Mock VS MBV6h (**b**), Mock VS MBV12h (**c**), and Mock VS MBV24h (**d**). Red dots (Up) represent significantly up-regulated genes (*P* < 0.01, fold change ≥2); Yellow dots (Up) represent extremely significantly up-regulated genes (*P* < 0.05, fold change ≥2); Mazarine dots (Down) represent significantly down-regulated genes (*P* < 0.01, fold change ≤0.5); Wathet dots (Down) represent extremely significantly downregulated genes (*P* < 0.05, fold change ≤0.5); black dots (nosig) represent insignificantly differential expressed genes

To investigate changes in gene expression profiles induced by BVDV infection, FPKM expression values of the genes were calculated based on the read counts using featureCounts software. The FC values of each gene at different time post infection compared with control group were also calculated using DESeq2 software. The threshold values FDR ≤ 0.05 and |Log2FC| ≥ 1 were used to identify DEGs between groups at different time points post-infection relative to the control group. Differential expression analysis identified 1297, 1732, 3072, and 1877 DEGs in different comparison groups Mock vs. MDBK cells infected with BVDV post 2 h(MBV2h), Mock vs. MBV6h, Mock vs. MBV12h, Mock vs. MBV24h post-BVDV infection (Fig. 2, Additional file 2: Figure S5 and Additional files 3, 4, 5, 6, 7, 8, 9). Differential expression analysis for the comparison groups, Mock vs. MBV2h, Mock vs. MBV6h, Mock vs. MBV 12 h, and Mock vs. MBV24h, identified 743, 1068, 1863, and 1035 upregulated genes and 554, 665, 1342, and 853 downregulated genes, respectively (Fig. 2).

During BVDV replication, double-stranded RNA (dsRNA) are generated to facilitate multiplication of progeny virus. dsRNA is an important pathogen-associated molecular pattern (PAMP) of BVDV that activates the host innate immunity. In our previous study, we detected the dynamic changes that occur in the BVDV negative strand in MDBK cells infected with 10 MOI BVDV using strand specific real-time PCR (Additional file 2: Figure S6). The negative strand of BVDV RNA in the host cells was detected at 2 h post-infection, and the amount of the negative strand of BVDV increased from 2 h to 6 h after BVDV infection. However, the amount of the negative strand of BVDV decreased at 12 h and gradually increased after 12 h. In addition, all biological processes in the life cycle of BVDV could be observed during the logarithmic growth phase. The DEGs identified in the comparison groups Mock vs. MBV2h, Mock vs. MBV6h, Mock vs. MBV12h, and Mock vs. MBV24h can help understand the mechanisms underlying the host response to BVDV infection. A total of 24 DEGs were identified, including *ACLY*, *HMGCS1*, *INSIG1*, *LPIN1*, *HMGCR*, and *ACSS2* in group MBV2h vs. MBV6h (Additional file 7). The genes *ACLY*, *HMGCS1*, *INSIG1*, *LPIN1*, *HMGCR*, and *ACSS2* were found to be significantly upregulated in comparison groups Mock vs. MBV6h, Mock vs. MBV 12 h, and Mock vs. MBV24h groups.

### Enrichment analysis of GO terms and KEGG pathway

**Fig. 3:**
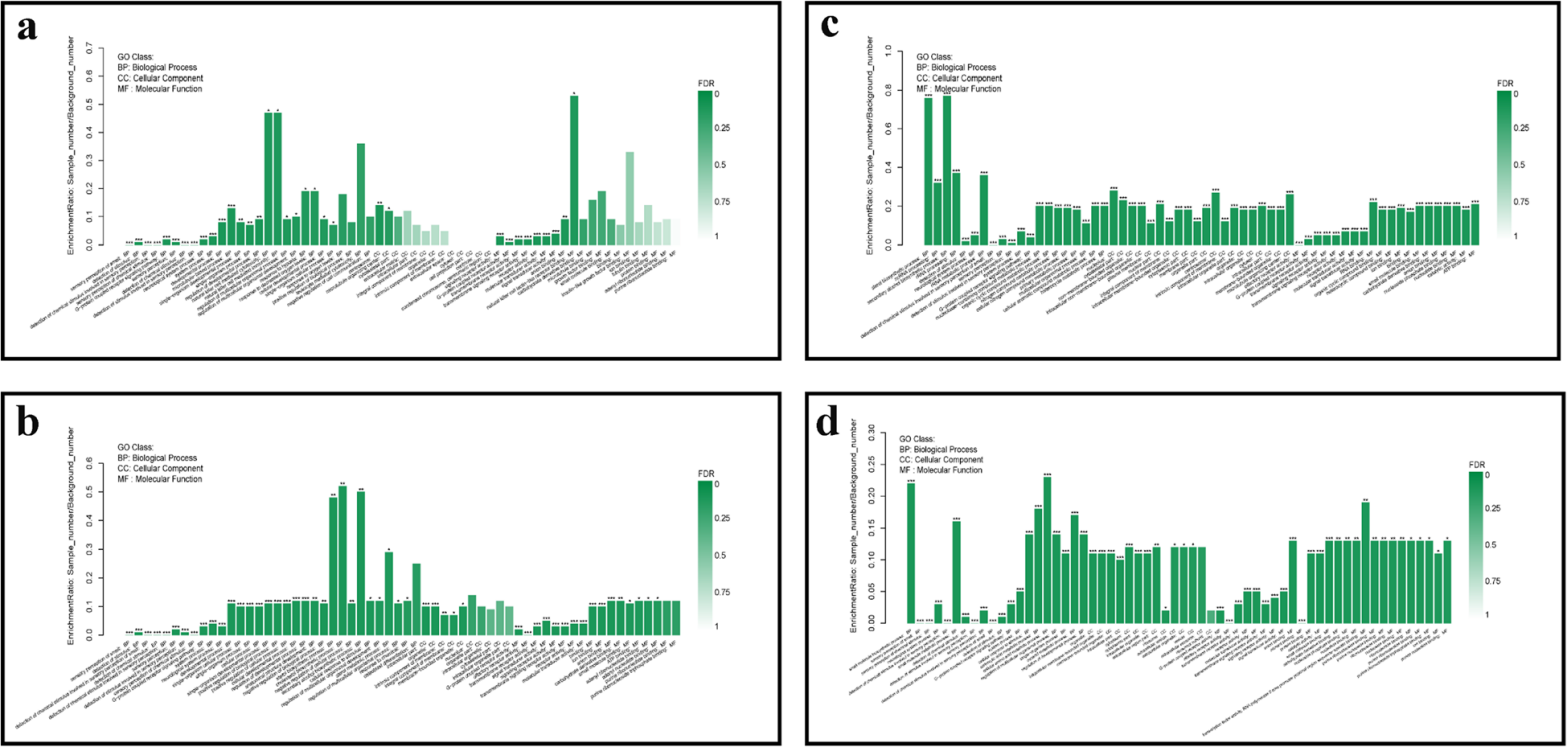
GO enrichment analysis of DEGs in different comparison groups, Mock VS MBV2h (**a**), Mock VS MBV6h (**b**), Mock VS MBV12h (**c**), and Mock VS MBV24h (**d**). GO terms are on the x axis. Enrichment ratio of genes shown as GO terms for BP, CC, and MF. * means GO categories with significant enrichment

Compared with general description for properties of gene or transcripts with functional annotation, the lowest level of gene function and KEGG pathway can be annotated by enrichment analyses. In addition, through fisher test, significantly enriched gene function and KEGG pathway of DEGs will be determined. The most detail information on gene function and KEGG pathway provided by enrichment analyses which facilitate us to screen the unique insights on the response to BVDV-infection. Enrichment analyses of GO terms and KEGG pathways were performed for DEGs using GOatools and KOBAS, respectively. GO terms and KEGG pathways that satisfy the corrected *p*-value ≤0.05 were considered significantly enriched. The enriched GO terms were ordered based on the three categories, including biological processes (BP), cellular components (CC), and molecular function (MF) (Fig. 3). DEGs in comparison group Mock vs. MBV2h were primarily associated with GO terms related to stimulation, G protein-coupled receptor signalling pathway, transmembrane signalling transduction, such as sensory perception of smell, detection of stimulus, sensory perception, G-protein coupled receptor signalling pathway, G-protein coupled receptor activity, transmembrane signalling receptor activity (Fig. 3a). The majority of DEGs in comparison group Mock vs. MBV6h were associated with stimulation-related biological processes, cellular processes, and signal transduction-related molecular functions, such as detection of stimulus, G-protein coupled receptor signalling pathway, positive regulation of biological process/cellular process, negative regulation of biological process/cellular process, G-protein coupled receptor activity, signal transducer activity, signalling receptor activity, and transmembrane signalling receptor activity(Fig. 3b). The majority of DEGs in comparison group Mock vs. MBV12h were assigned to biological processes associated with stimulus, biosynthesis and metabolism, cellular components (cytoskeletal part, membrane part, cytoplasm), and molecular functions, such as transmembrane receptor activity, transmembrane signalling receptor activity, RNA binding, binding, ATP binding, protein binding (Fig. 3c and Additional file 10). The majority of DEGs in comparison group Mock vs. MBV24h were associated with the regulation of biological processes, cellular processes, and signal transduction, such as small molecule biosynthetic process, sensory perception of smell, regulation of signalling, positive regulation of biological process, positive regulation of cellular process, regulation of response to stimulus, G-protein coupled receptor activity, transmembrane receptor activity, signal transducer activity, protein binding, and small molecule binding (Fig. 3d and Additional file 11). DEGs identified from comparison group MBV2h vs. MBV6h were mainly assigned to biological processes associated with lipid synthesis and metabolism, such as lipid biosynthetic process, cholesterol biosynthetic process, secondary alcohol biosynthetic process, and lipid metabolic process (Additional file 2: Figure S7).

**Fig. 4:**
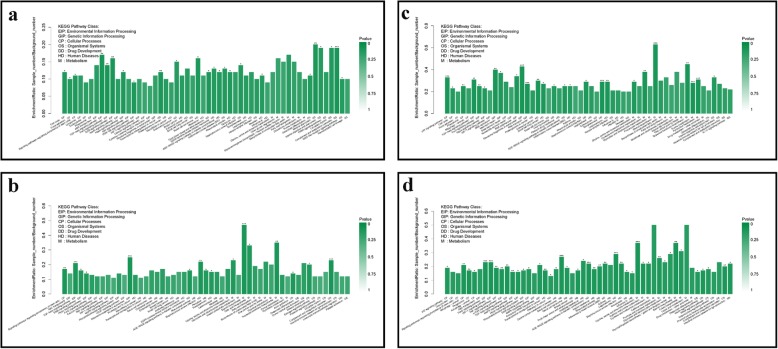
KEGG Pathway enrichment analysis of DEGs in different comparison groups, Mock VS MBV2h (**a**), Mock VS MBV6h (**b**), Mock VS MBV12h (**c**), and Mock VS MBV24h (**d**). The name of KEGG pathway are on the x axis. Enrichment ratio of genes shown as the name of KEGG pathway for seven categories, environmental information processing (EIP), genetic information processing (GIP), cellular processing (CP), organismal systems (OS), drug development (DD), Human diseases (HM), Metabolism (M). * means KEGG pathway with significant enrichment

The genes in an organism act in a coordinated manner to perform their biological functions. Pathway analysis can help better understanding the biological functions of genes. Enrichment analysis of KEGG pathways associated with the DEGs can be used to determine the biochemical metabolic and signal transduction pathways. The analysis identified 24, 16, 27, and 44 KEGG pathways that were significantly enriched in DEGs in comparison groups Mock vs. MBV2h, Mock vs. MBV6h, Mock vs. MBV12h, and Mock vs. MBV24h, respectively (Fig. 4). The significantly enriched pathways in DEGs from comparison group Mock vs. MBV2h mainly included the complement and coagulation cascades, TGF-beta signalling pathway, FoxO signalling pathway, ErbB signalling pathway, PI3K-Akt signalling pathway, and signalling pathways associated with pathogen-infection. The pathways significantly enriched in DEGs from comparison group Mock vs. MBV6h mainly included complement and coagulation cascades, steroid biosynthesis, TGF-beta signalling pathway, and fatty acid biosynthesis. The significantly enriched pathways in DEGs from comparison group Mock VS MBV12h mainly included steroid biosynthesis and p53 signalling pathway (Additional file 12). The pathways that were significantly enriched in the DEGs from comparison group Mock vs. MBV24h mainly included amino acid metabolism, MAPK signalling pathway, HIF-1 signalling pathway, TNF signalling pathway, and mTOR signalling pathway (Additional file 13). KEGG pathways which were significantly enriched in all comparison groups included complement and coagulation cascades, TGF-beta, FoxO, ErbB, Hippo, and AGE-RAGE signalling pathways in diabetic complications. The PI3K-Akt, NOD-like receptor, HIF-1, MAPK, TNF, and mTOR signalling pathways play important roles in the recognition of pathogen-associated molecular patterns, signalling transduction, and the regulation of host immune system. These results provided important insights on the interactions between BVDV and host.

### Protein-protein interaction network (PPI) analysis of DEGs

To further understand the biological relevance of DEGs, PPI network analyses of DEGs was performed using STRING database. The PPI protein-protein network was visualized using NetworkX package in Python.

**Fig. 5:**
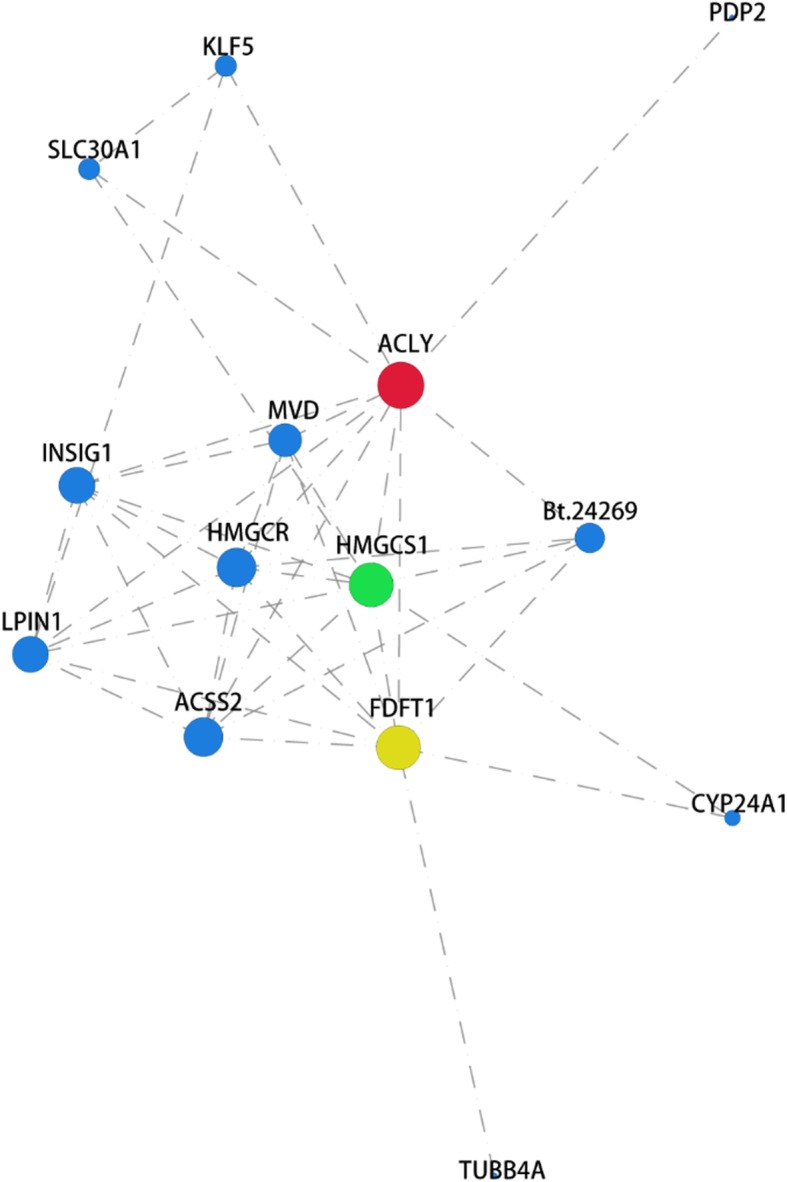
Protein-protein interaction network analysis of DEGs in group MBV2h VS MBV6h. The STRING database (http://string-db.org/) was used to analyse the protein-protein interaction network based on the proteins corresponding to selected DEGs. Protein interaction relationship of selected DEGs existing in the database were extract for the construction of network. Then, the visualization of the network is carried out using network X under Python. The size of the node is proportional to node degree. ACLY, HMGCS1, FDFT1, were the three genes with the most node degree in the analysis, labelled in red blot, green blot and yellow blot, respectively

PPI network analyses of DEGs in groups Mock vs. MBV2h and Mock vs. MBV24h were performed based on DEGs with FDR ≤ 0.05 and |Log2FC| ≥ 2. PPI network analyses identified a total of 233 interaction relationships among 89 genes within the DEGs from comparison group Mock vs. MBV2h (Additional file 2: Figure S9). The DEGs *OAS1Y*, *FOS*, *EGR1*, and *PAI2* were found to play important roles in maintaining the tight connection of the whole network. The analysis additionally identified a total of 172 interaction relationships among 46 genes within the DEGs from comparison group Mock vs. MBV24h (Additional file 2: Figure S10). The DEGs *OAS1Y*, *CDKN1A*, *FOSB*, *NOTCH3*, *FLT3*, *HDAC5*, *RELB*, and *CsDKN2B* were found to play important roles in maintaining the tight connection of the whole network. Furthermore, 41 interaction relationships were identified among 14 genes in DEGs comparison group MBV2h vs. MBV 6 h (Fig. 5). The DEGs *ACLY*, *HMGCS1*, *FDFT1*, *HMGCR*, and *ACSS2* were found to play important roles in maintaining the tight connection of the whole network. The network centrality coefficients of the genes are shown in Additional file 1: Table S3-S5. The DEGs *OAS1Y*, *FOS/FOSB*, *EGR1*, *PAI2*, *CDKN1A*, *NOTCH3*, *FLT3*, *HDAC5*, *RELB*, *CDKN2B* play key roles in multiple cellular activities (including cell cycle, apoptotic process, regulation of gene expression, and disease development) and signal transduction. The above results indicated that BVDV interferes with the expression of these genes to promote its own replication in host cells. The DEGs *ACLY*, *HMGCS1*, *FDFT1*, *HMGCR*, and *ACSS2* were associated with lipid synthesis and metabolism, thereby suggesting that BVDV infection induced lipid synthesis and metabolism activity in host cells.

### Quantitative real-time PCR (RT-qPCR) analysis

**Fig. 6:**
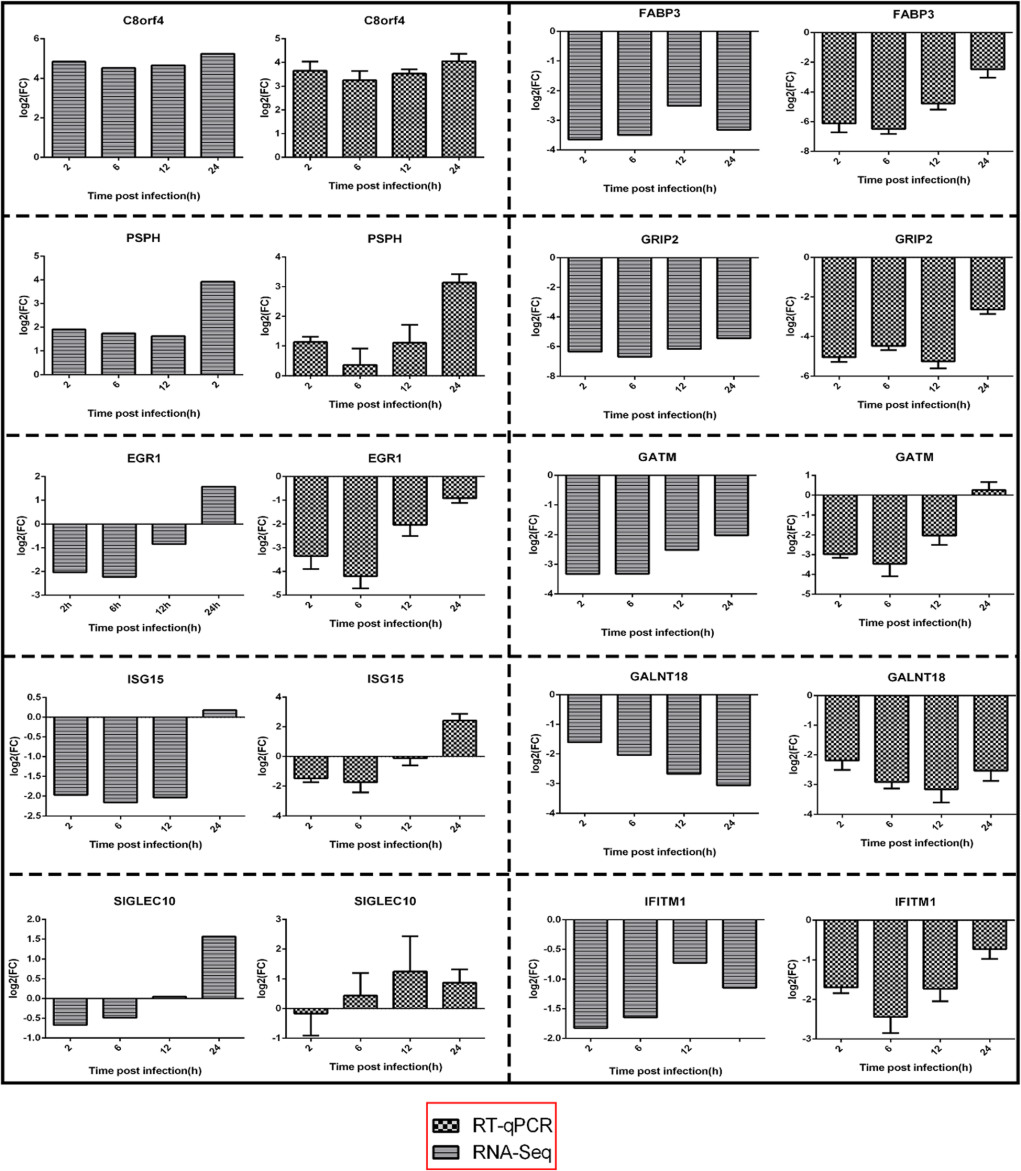
Expression level of ten randomly selected gene validated by RT-qPCR. β-actin gene was used as an internal control and relative quantity of gene expression (fold change) of each gene was calculated with the comparative 2^-ΔΔCT^ method. Values (RT-qPCR) shown were mean with SD

**Fig. 7:**
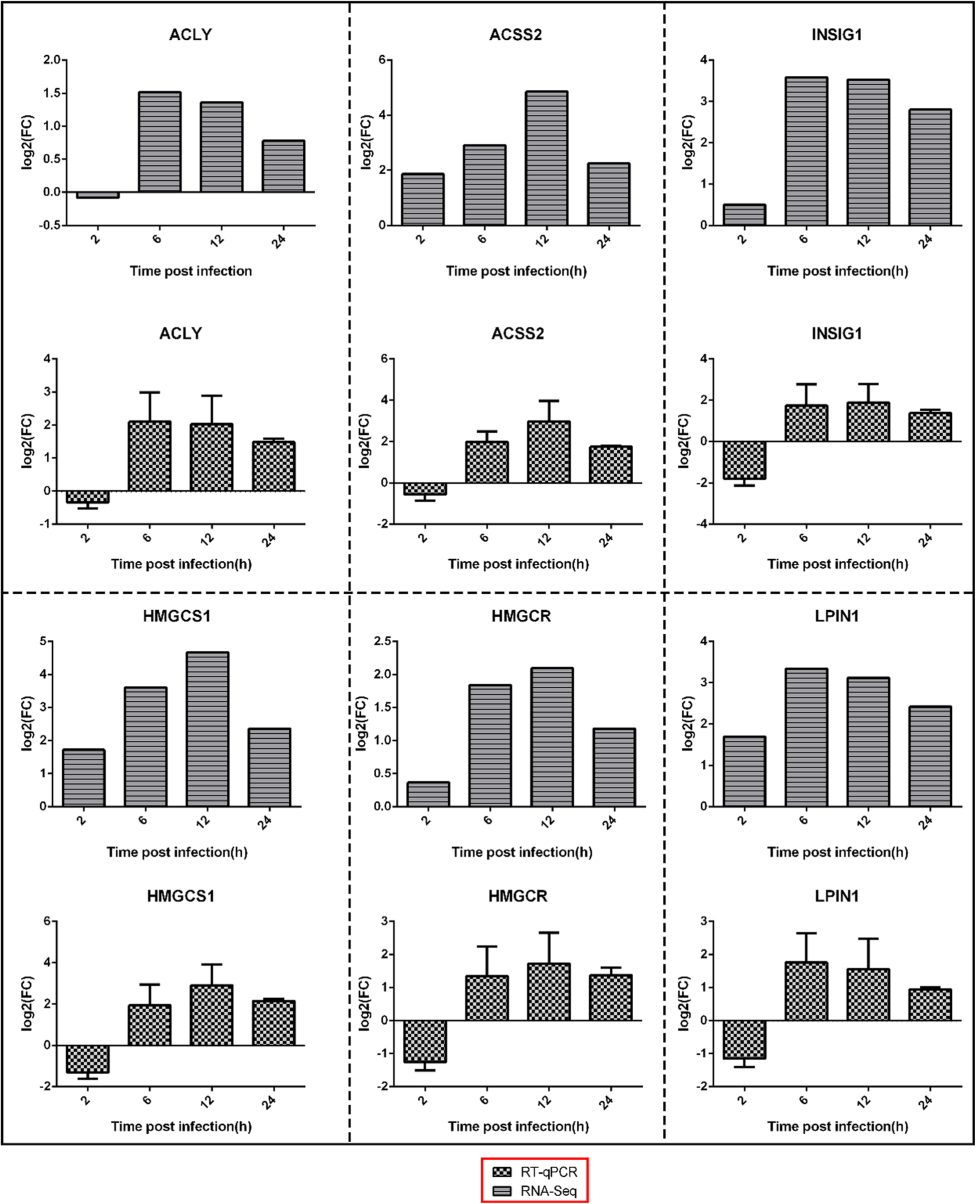
Expression level of genes, ACLY, ACSS2, INSIG1, HMGCR, HMGCS1, LPIN1, validated by RT-qPCR. β-actin gene was used as an internal control and relative quantity of gene expression (fold change)of each gene was calculated with the comparative 2^-ΔΔCT^ method. Values (RT-qPCR) shown were mean with SD

**Fig. 8:**
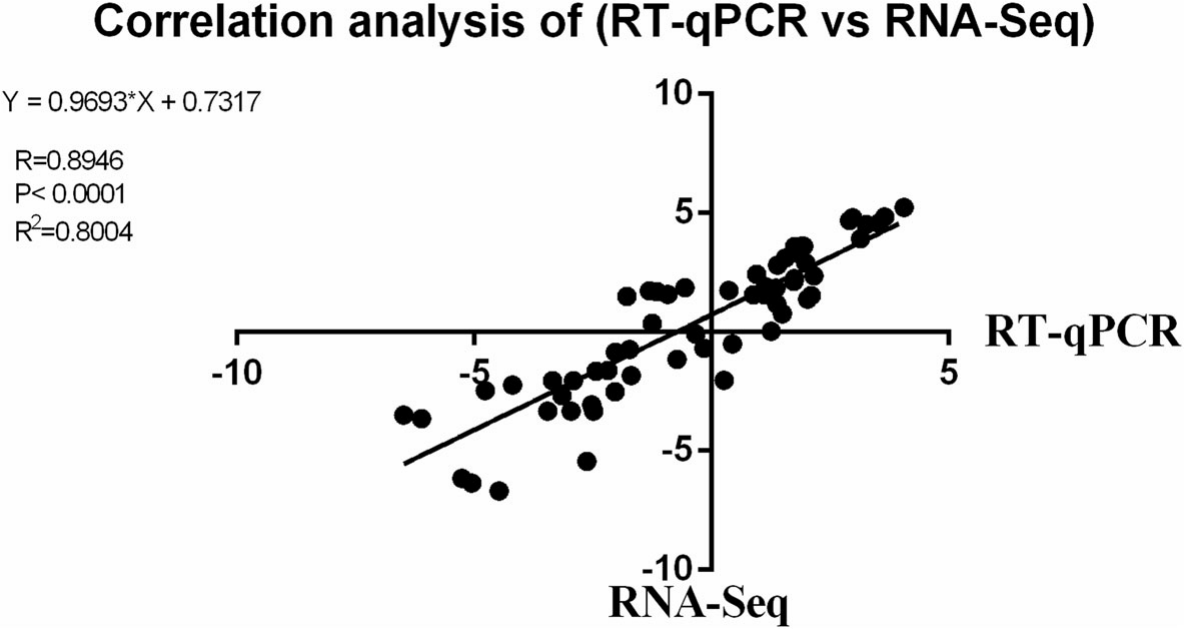
Correlation of fold change analysed by data obtained using RT-qPCR (x axis) with RNA-Seq platform (y axis). Correlation analysis was performed using GraphPad software 6.0 (San Diego, CA)

To validated the reproducibility and repeatability of DEGs identified from transcriptome sequencing, we randomly selected ten genes, namely, *C8orf4*, *PSPH*, *ISG15*, *EGR1*, *SIGLEC10*, *FABP3*, *GRIP2*, *GALNT18*, *GATM*, and *IFITM1*, for RT-qPCR analysis (Fig. 6). The results showed that these genes were significantly differentially expressed and were consistently upregulated or downregulated with the gene expression changes based on RNA-Seq, thereby suggesting that the DEGs obtained from transcriptome sequencing were reliable. The genes *ACLY*, *ACSS2*, *INSIG1*, *HMGCR*, *HMGCS1*, and *LPIN1* were also validated by RT-qPCR (Fig. 7). The trends in differential expression of these genes confirmed by RT-qPCR in the Mock vs. MBV6h, Mock vs. MBV 12 h, and Mock vs. MBV24h groups were consistent with those of the transcriptome sequencing results. However, the genes *ACSS2*, *INSIG1*, *HMGCR*, *HMGCS1*, and *LPIN1* were downregulated in the Mock vs. MBV group at 12 h, which was inconsistent with the transcriptome analysis. Correlation was measured by scatterploting log2 fold changes between RNA-Seq and RT-qPCR. As shown in Fig. 8, high correlation was observed between the results from RT-qPCR and RNA-Seq, with the correlation coefficient (R^2^) as high as 0.8004.

## Discussion

BVDV is responsible for the severe economic losses because of decreased production performance, slow foetal growth, diarrhoea, respiratory symptoms, reproductive dysfunctions, immunosuppression, concurrent infections, and persistently infected (PI) cattle [29, 30]. BVDV employed various strategies to evade the innate immune system. More in-depth studies on BVDV-host interactions will be of great importance in elucidating the mechanisms underlying the pathogenesis and the progression of persistent BVDV infection.

RNA-Seq is a recently developed high-throughput approach for transcriptome profiling that achieves considerably more precise measurement of the expression levels of transcripts and their isoforms compared to other methods [31]. The mechanisms responsible for the pathogenesis and the formation of persistent BVDV infection are complex, and RNA-Seq provides an effective means for the systematic study of host response to viral infection and for investigating BVDV and host interactions. In the present study, transcriptomes of MDBK cells inoculated with BVDV at the early stage of infection were sequenced using the NovaSeq 6000 platform. DEGs were identified in MDBK cells infected with BVDV at 2, 6, 12, and 24 h post-infection. The expression changes of randomly selected genes, which was analysed by RT-qPCR, showed a strong correlation with that identified with RNA-Seq. This supported the reliability of data from RNA-Seq analysis.

Metabolism provides the energy and materials required for all biological processes [32, 33]. Viruses are obligate parasites that derive all energy and materials necessary for infection and replication from the host cell [32, 34, 35]. Some viruses generate a suitable intracellular microenvironment for the viral life cycle by altering the host’s cellular metabolic network [36]. In the present study, analysis of the DEGs revealed that several key genes that were upregulated during the BVDV life cycle; these genes included *ACLY*, *HMGCS1*, *HMGCR*, *ACSS2*, *INSIG1*, and *LPIN1*, which are related to lipid metabolism. The *LPIN1* gene, which encodes the lipin-1 protein, plays critical roles in adipocyte differentiation and lipid metabolism [37, 38]. Lipin-1 can bidirectionally regulate fat metabolism and exerts significant effect on fat deposition, which are potentially correlated with animal carcass traits and meat quality traits [39–43]. The *INSIG1* gene primarily affects lipid metabolism by regulating sterol regulatory element binding protein (SREBP) and 3-hydroxy-3-methylglutaric coenzyme A reductase (HMGCR) and is correlated with economic traits of livestock [44–48]. The *ACLY* gene is related to intramuscular fat deposition in cattle, which is of great significance for beef quality improvement [49, 50]. Other genes, such as *HMGCS1*, *HMGCR*, and *ACSS2*, were also associated with growth performance and meat quality improvement in cattle [51–56]. The dysregulated expression of these genes can also be associated with the development of disease, such as cancers, viral infection, and diabetes mellitus [34, 35, 57–60]. Previous studies confirmed that positive-strand RNA viruses and enveloped viruses rewire lipid metabolism for replication [34, 61, 62]. In the present study, the genes that were upregulated during BVDV infection were related to lipid metabolism, thereby suggesting that BVDV might alter host lipid metabolism to facilitate itself survival. This phenomenon was similar to that of hepatitis C virus (HCV) infection in hepatocytes, in which tumour-like glutamine metabolism is induced to create an environment that is favourable for viral replication [61]. The above findings may provide a reasonable explanation for the low performance of cattle with persistent infections. However, further in vivo and in vitro validation are required.

Innate immunity is the first line of defence against pathogen invasion. Interferon-stimulated genes (ISGs) are important components of the host innate immune system that are instrumental in the defence mechanism against viral infection [63]. ISGs that were found to be differentially expressed during BVDV infection are interesting. *OAS1Y*, *Mx1*, and *ISG15*, which have direct antiviral activity, were found to be downregulated during BVDV infection. 2′-5’oligoadenylate synthase (OAS), a member of the double-stranded RNA family, is an important antiviral protein induced by IFN and is involved in cellular innate immune defence [64]. OAS protein can be activated by replication intermediate dsRNA, and activated OAS can catalyse the synthesis of 2′-5′ oligoadenylate (2′-5’A) [65]. Next, RNAse L is activated by 2′-5’A. Viral RNA is degraded by RNAse L. In a previous study, dynamic changes in the negative-strand RNA of BVDV during replication in MDBK cells were detected by strand-specific reverse transcription real-time quantitative PCR (ssRT-qPCR). The findings showed that the abundance of the negative-strand RNA of BVDV initially increased between the 2 h and 6 h time points, decreased between the 6 h and 12 h time points, and then gradually increased between the 12 h and 36 h points. Peak levels of the negative-strand RNA of BVDV were observed between 36 h and 48 h after infection of MDBK cells with BVDV (Additional file 2: Figure S6). The observed downregulation of the negative-strand RNA levels between the 6 h and 12 h time points may be associated with the degradation of dsRNA by the machinery of the host innate immune defence system, such as the OAS/RNAse L system. The ISG15 protein, which is encoded by IFN-stimulated gene 15, is a ubiquitin-like modified protein. ISG15 protein and its ubiquitin-like modification system participate in the innate immune response and are crucial to facilitate the antiviral effects of interferons [66]. Mx proteins, interferon (IFN)-induced GTPases, which exert potent antiviral activity against various single-stranded RNA viruses, are members of dynamin superfamily. Mx proteins inhibit viral replication through diverse strategies. Mx protein can inhibit virus replication by lysing ribonucleoprotein complexes, isolating viral nucleocapsid proteins in large membrane-associated perinuclear complexes, and inhibiting viral primary transcription [67–70]. The downregulated expression of virus resistance genes could be part of the mechanisms by which BVDV escapes from the host immune defence system, which is mediated by inhibiting the production of IFN in host cells.

The recognition of PAMPs and the activation of signalling transduction pathways are the basis for the activation of host immune defence system. The molecular components that participate in signal transduction pathways that activate the host immune defence system are blocked by pathogens. Enrichment analysis of KEGG pathways of DEGs will help understand the signal transduction pathways that are activated or inhibited by BVDV infection and provide clues into the interactions between BVDV and host. KEGG pathway enrichment analysis revealed that the DEGs from all treatment groups were enriched in pathways involved in complement and coagulation cascades, TGF-beta, FoxO, and AGE-RAGE signalling pathways in diabetic complications, and ErbB and Hippo signalling pathway [71–73]. The complement system, because of its dual effector and priming functions, is a mediator of innate immunity and acts as a nonspecific defence mechanism against pathogens. The complement system is composed of more than 35 soluble plasma proteins that play essential roles in innate and adaptive immunity [74]. A total of 11 DEGs were identified in this study, namely, *F3*, *C1R*, *KNG1*, *CLU*, *C3*, *FB*, *SERPINA5*, *SERPINE1*, *C1S*, *F2RL2*, *C*, which were enriched in pathways related to the complement and coagulation cascades and were downregulated during BVDV infection. C1, which is composed of C1q, C1r, and C1s, is the protein complex that initiates the classical pathway of complement activation [75]. Complement factor B (FB) participates in the formation of C3 convertase [76, 77]. The downregulated expression of the genes *C1* and *FB* can inhibit the activation of complement system. The above findings suggested that BVDV inhibits the complement system and plays an important role during the early stages of BVDV infection. The inhibition of complement activation during viral infection has been confirmed in previous studies. Complement activation is known to be inhibited by HCV NS3/4A protease [78]. In the acute stage of rotavirus virus (RV) infection, *C3* expression is significantly downregulated; C3 levels are restored to normal levels during the convalescent stage, suggesting that C3 participates in inflammatory immunity in the early stage of RV infection [79]. Signalling pathways, including TGF-beta, FoxO, and AGE-RAGE signalling pathways in diabetic complications, and ErbB and the Hippo signalling pathways play important roles in the regulation of cell proliferation, differentiation, apoptosis, immune function, and the development of disease [80–87]. KEGG pathways, such as PI3K-Akt and NOD-like receptor signalling pathways, were significantly enriched in DEGs in the Mock vs. MBV2h after BVDV infection, as well as the pathways HIF-1, MAPK, TNF, and mTOR signalling pathway, which were significantly enriched in DEGs in Mock vs. MBV24h after BVDV infection, also regulate cell progression, including the recognition of pathogen-associated molecular patterns (PAMP), signalling transduction, the regulation of host immune system regulation [88–92]. Our results indicated that these signalling pathways are closely related to BVDV infection. Further investigation of these enriched signalling pathways will help further understand BVDV infection and pathogenesis, identify targets for drug development, and contribute to the prevention and treatment of bovine viral diarrhoea.

## Conclusion

In present study, we characterized the transcriptome profile of MDBK cells infected with BVDV. The dynamic changes of differentially expressed genes of BVDV infection contributed to understanding the molecular mechanisms of BVDV-host interaction. Our findings suggested that BVDV-infection induced altering the host’s metabolic network, the inhibition of the expression of antiviral proteins and genes within the complement system might be contributed to BVDV proliferation. The above findings provided unique insights for further studies on the mechanisms underlying BVDV-host interactions and could serve as the basis for the prevention and treatment of bovine viral diarrhoea.

## Methods

### Ethic statement

No animal experiment was involved in this study.

### Virus and cells

Madin-Darby bovine kidney (MDBK) cells were cultured in Dulbecco’s modified eagle medium (DMEM) (Gibco) supplemented with 8% fetal bovine serum (FBS) (Gibco). MDBK cells were maintained at 37 °C in an atmosphere with 5% CO_2_. BVDV BJ-2016 strain is a NCP BVDV isolated from the commercial bovine fetal serum. The virus titer was detected by indirect immunofluorescence assay (IFA).

### Growth curves of BVDV

The growth curves of BVDV at different multiplicity of infection (MOI) were done. Briefly, MDBK cells (2 × 10^6^ cells/well) were plated into 6-well cell culture plate and cultured in DMEM with 8% FBS. After 12 h, MDBK cells were inoculated with 0.1, 1, 10 MOI BVDV, respectively. Three biological repeats were set for each titer. The cell supernatants were collected at 2 h, 6 h, 12 h, 24 h, 36 h, 48 h post-infection, separately. The viral genome was quantified by reverse transcription real-time quantitative PCR (RT-qPCR) [93]. Number of viral genomes per milliliter of cell supernatant was calculated according to the standard curve.

### Sample collection and RNA extraction

MDBK cells were plated onto T25 cell culture flasks and grown in DMEM containing 8% FBS. After 12 h, MDBK cells were inoculated with 10 MOI BVDV and maintained at 37 °C with 5% CO_2_ atmosphere for 1 h. Afterwards, cells were washed thrice with PBS, and grown in DMEM without serum. Samples were collected at 2, 6, 12, and 24 h post-infection based on the BVDV growth curves. The experiment was performed with three biological replicates for error reduction.

Total RNA was extracted using TRIzol® Reagent (Invitrogen) according to the manufacturer’s instructions. Then, the quality and integrity of the total RNA were assessed using an Agilent 2100 Bioanalyzer (Agilent Technologies, Santa Clara, CA) and 1.2% agarose gel electrophoresis; the RNA concentration was measured using a NanoDrop 2000 instrument (NanoDrop Technologies, Technologies, Wilmington, DE). High-quality RNA samples with OD260/280 ratios ranging from 1.8-2.2 and OD260/230 ≥ 2.0, RNA integrity number (RIN) ≥8, and total RNA concentration ≥ 50 ng/μL were used for library preparation.

### Library preparation and Illumina NovaSeq 6000 sequencing

RNA-Seq libraries were prepared using the Illumina TruSeq™ RNA sample preparation Kit (Illumina, San Diego, CA). Briefly, oligo (dT) magnetic beads were used to enrich for mRNAs, which contain poly A tails, from the total RNA. The enriched mRNAs were cleaved into 300-bp fragments using fragmentation buffer. The cleaved mRNA fragments served as the templates for double-stranded cDNA (dscDNA) synthesis using the SuperScript dscDNA synthesis kit (Invitrogen, CA) following the manufacturer’s instructions. The synthesized dscDNA was subjected to end-repair and A-tailed. The indexed adapters were ligated to the A-tailed dscDNA. The dscDNA with indexed adapters were purified and enriched by PCR for 15 cycles. The quantity and quality of enriched dscDNA were assessed using TBS380 and NanoDrop 2000 spectrophotometer and Agilent 2100 Bioanalyzer. The libraries were then used for paired-end (PE) sequencing with the Illumina NovaSeq 6000 platform.

### Quality control and read mapping

Quality control of the reads generated from RNA-Seq was performed using SeqPrep (https://github.com/jstjohn/SeqPrep) and Sickle (https://github.com/najoshi/sickle). Adapter and primer sequences were removed, and sequences with lengths below 20 bp were discarded. After removing adapter and primer sequences, low-quality bases were trimmed from the 3′ end of the reads. After trimming low-quality bases, sequences with quality values less than 10 were discarded. Sequences with N ratios higher than 10% were also removed. The error rate (%), Q20 and Q30 values, GC-content (%), and sequence duplication levels of the resulting high-quality clean reads were then evaluated.

The high-quality reads were mapped to the reference *Bos taurus* genome (UMD3.1) (http://www.ensembl.org/Bos_taurus/Info/Index) using Hisat2 (http://ccb.jhu.edu/software/hisat2/index.shtml) and the reference-based assembly of transcripts was performed using Stringtie (http://ccb.jhu.edu/software/stringtie/). By comparing with the reference *Bos taurus* genome, on the one hand, genes with annotation information can be identified in assembled genome. On the other hand, transcripts without annotated information can be obtained, which are defined as new transcripts. Novel transcripts and genes were predicted by gffcompare software. And then, the gene prediction was used for calculation of gene expression values.

### Functional annotation and classification

For functional annotation and classification, all transcripts and their corresponding genes were compared with the Clusters of Orthologous Groups of proteins (COG, http://www.ncbi.nlm.nih.gov/COG/), Gene Ontology (GO, http://www.geneontology.org) and Kyoto Encyclopedia of Genes and Genomes (KEGG, http://www.genome.jp/kegg/) databases. GO analysis was conducted using the BLAST2GO software with default parameters. COG functional classification was conducted using Blastx software in the STRING database. KEGG pathway annotation was performed using KOBAS (http://kobas.cbi.pku.edu.cn/).

### Differential expression analysis

Read counts of each transcript or gene were calculated using featureCounts software. Gene expression values were expressed as reads per kilobase of exon per million fragments mapped (FPKM) using featureCounts software. To identified true differentially expressed genes (DEGs), the false discovery rate (FDR) was used for the rectification of the *p*-values. The fold change (FC) of each transcript or gene in different groups was calculated. Transcripts or genes with FDR ≤ 0.05 and |Log2FC| ≥ 1 were considered as significant DEGs. DESeq2 was used to perform differential expression analysis of genes or transcripts.

The DEGs were subjected to enrichment analyses of GO and KEGG pathways. Fisher’s exact test and multiple correction method, including Bonferroni, Holm, Sidak, and false discovery rate, were used for the enrichment analyses to correct p-values and the false positive rate. Functional enrichment analysis was performed using GOatools. GO terms with corrected *p*-value ≤0.05 were considered as significantly enriched. Enrichment analysis of KEGG pathways was conducted using KOBAS software. KEGG pathways with corrected *p*-value ≤0.05 were considered as significantly enriched.

Protein-to-protein interaction network analyses of DEGs was performed using STRING database (http://string-db.org/) and visualized the protein-protein interaction network relationship using NetworkX within Python.

### Validation of transcriptome sequencing results

Reverse transcription quantitative real-time PCR (RT-qPCR) was performed to validated the DEGs identified from transcriptome sequencing. Ten randomly DEGs, namely, *C8orf4, PSPH, ISG15, EGR1, SIGLEC10, FABP3, GRIP2, GALNT18, GATM, IFITM1,* were selected for RT-qPCR validation. The gees *ACLY, ACSS2, INSIG1, HMGCR, HMGCS1, LPIN1*, were additionally quantified by RT-qPCR for analyses of genes expression. Total RNA extraction was performed using TRIzol reagent following the manufacturer’s instructions. cDNA synthesis was conducted using the TransScript One-Step gDNA Removal and cDNA Synthesis SuperMix (TransGen Biotech, Beijing, China). Sequence-specific primers of the randomly selected genes were designed using Primer Express software (Table 2). ChamQ™ Universal SYBR qPCR Master Mix (Vazyme Biotech Co., Ltd) was used to perform RT-qPCR following the manufacturer’s instructions. RT-qPCR was performed in 20-μL reaction volumes containing 10 μL of SYBR qPCR Master Mix, 0.8 μL of upstream and downstream primers (10 μM), 1 μL of cDNA template, and 7.4 μL of ddH_2_O. The following reaction profile was used: 95 °C for 30 s, followed by 40 cycles of 95 °C for 5 s and 60 °C for 30 s; melting curve analysis was performed to validate specific amplification. β-actin gene was used as an endogenous reference gene. RT-qPCR was performed in a 96-well plate on an ABI QuantStudio 7 Flex real-time system (Applied Biosystems, Foster City, CA). The detection was performed in triplicate for each biological replicate. The relative expression values of selected genes were calculated using the 2^-ΔΔCt^ method and normalized against the expression levels of the β-actin gene.

**Table 2:** Primers for RT-qPCR in this study

Name	Primer sequence	Name	Primer sequence
C8orf4-F	CCATCCACGGCTACCACTTC	GATM-F	CATTGGGCCTGGTCTTGTG
C8orf4-R	AGCCTCTGTAATGCTTCTTGGTCTA	GATM-R	ATGGTCCATCCTGCTTTCTTGA
PSPH-F	TCAAGGCTGCCCTCACACA	IFITM1-F	CTACCGCCAAGTGCCTGAA
PSPH-R	AGGAGCCTCTGCACCTGTTC	IFITM1-R	AATGAGAAGAACGATCGATCCAA
ISG15-F	TCGCCCAGAAGATCAATGTG	HMGCS1-F	GGCTCCCTGGCTTCTGTTC
ISG15-R	AGCACCTCCCTGCTGTCAAG	HMGCS1-R	AGAAAACACGCCGATCCTCTT
EGR1-F	GGCCGAGATGCAGCTGAT	INSIG1-F	CCTCGCCACCCTGATCAC
EGR1-R	TGTCCATGGTGGGCGAAT	INSIG1-R	CACGCCTCCCGAGAAGAAG
SIGLEC10-F	CAGCTTACGGCTTCTGGTTCA	ACSS2-F	CCGTCTGCTCATGAAGTTTGG
SIGLEC10-R	TTGTGTCCACGTCCTGATCTG	ACSS2-R	TTGATAGGTTCGCCCACAGTT
FABP3-F	GACAGGAAAGTCAAGTCCATCGT	HMGCR-F	CTGCTGCTGGTCGACCTTTC
FABP3-R	CCCGCACAAGTGATGTCTCTT	HMGCR-R	TCTCCCTCACTTCATCCTGTGA
GRIP2-F	CCTGGTCATCTCCGACATCAA	LPIN1-F	TGTTCAGTGTCACCACGCAGTA
GRIP2-R	ATTTGCACAGCGTCCTCCAT	LPIN1-R	GGTCCCATCAATGTCAGAGATGA
GALNT18-F	GTGCCGCAACCTCTCGTT	ACLY-F	CCAGAGGTAGACGTGCTAATCAAC
GALNT18-R	CACGGAGAGTGCTTCATTGACA	ACLY-R	TGAGCGTAATTCATGGTCTCCAT
β-actin-F	TCAGCAAGCAGGAGTACGATGA	β-actin-R	ATCCTGAGTCAAGCGCCAAA

## Supplementary information

**Additional file 1: Table S1.** Length distribution of transcripts. **Table S2.** The annotation of novel transcripts/genes compared with NR, Swiss-Prot, and Pfam databases. **Table S3.** Nodes centrality analysis of PPI network of group Mock vs. MBV2h. **Table S4.** Nodes centrality analysis of PPI network of group Mock vs. MBV24h. **Table S5.** Nodes centrality analysis of PPI network of group MBV2h vs. MBV6h.

**Additional file 2: Figure S1.** Venn analysis of functional annotation and classification within GO, COG and KEGG database. **Figure S2.** Functional annotation of the genes obtained from RNA-Seq. The different colours of the pie chart represent different GO terms, and the area represents the relative proportion of genes/transcripts in the GO Term. The number of genes were shown behind the subsets. Go terms of level 2 were annotated within the three Go categories, biological progress (BP), cellular progress (CC), and molecular function (MF). The functional annotation was performed using Blast2go. **Figure S3.** Function classification in Clusters of Orthologous Groups of Proteins (COG) of genes from RNA-Seq. Capital letters on x axis indicated the COG categories as listed on the right of histogram; y axis indicated the number of gene. **Figure S4.** Pathway annotation of genes from RNA-Seq. KEGG Pathway of level 2 within the seven categories of KEGG pathway as listed on the right of histogram were annotated. x axis indicated the number of genes annotated to the pathway; y axis indicated the name of the KEGG pathway. **Figure S5.** Venn analysis of differential expression genes from group Mock vs. MBV2h, Mock vs. MBV6h, Mock vs. MBV12h, Mock vs. MBV24h. **Figure S6.** Dynamic changes of BVDV RNA in MDBK cells infected with 10 MOI BVDV. a: BVDV positive strand RNA; b: BVDV negative strand RNA. Copies of BVDV positive or negative strand RNA was quantified by strand specific SYBR Green real-time PCR and shown as copies of positive or negative strand RNA of BVDV per microgram total RNA of cells. **Figure S7.** GO enrichment analysis of DEGs in comparison groups MBV2h vs. MBV6h(a), MBV6h vs. MBV 12 h(b), MBV12h vs. MBV24h(c). GO terms are on the x axis. Enrichment ratio of genes shown as GO terms for BP, CC, and MF. * means GO terms with significant enrichment. **Figure S8.** KEGG Pathway enrichment analysis of DEGs in comparison groups MBV2h vs. MBV6h (a), MBV6h vs. MBV12h (b), MBV12h vs. MBV24h (c). The name of KEGG pathway are on the x axis. Enrichment ratio of genes shown as the name of KEGG pathway for seven categories. * means KEGG Pathway with significant enrichment. **Figure S9.** Protein–Protein Interaction Network Analysis of DEGs with FDR ≤ 0.05 and |Log2FC| ≥ 2 in comparison group Mock vs. MBV2h. OAS1Y, FOS and EGR1 were three genes with the most node degree in the analysis, labelled in red blot, green blot and yellow blot, respectively. **Figure S10.** Protein–Protein Interaction Network Analysis of DEGs with FDR ≤ 0.05 and |Log2FC| ≥ 2 in comparison group Mock vs. MBV24h. OAS1Y and FOSB were the two genes with the most node degree, labeled in red blot and yellow blot, respectively.

**Additional file 3.** DEGs identified in comparison group Mock vs. MBV2h.

**Additional file 4.** DEGs identified in comparison group Mock vs. MBV6h.

**Additional file 5.** DEGs identified in comparison group Mock vs. MBV12h.

**Additional file 6.** DEGs identified in comparison group Mock vs. MBV24h.

**Additional file 7.** DEGs identified in comparison group MBV2h vs. MBV6h.

**Additional file 8.** DEGs identified in comparison group MBV6h vs. MBV12h.

**Additional file 9.** DEGs identified in comparison group MBV12h vs. MBV24h.

**Additional file 10.** Detail information of GO enrichment of DEGs in mock vs. MBV12h.

**Additional file 11.** Detail information of GO enrichment of DEGs in mock vs. MBV24h.

**Additional file 12.** Detail information of KEGG pathway enrichment of DEGs in mock vs. MBV12h.

**Additional file 13.** Detail information of KEGG pathway enrichment of DEGs in mock vs. MBV24h.

## Data Availability

The datasets generated and/or analyzed during the current study are available at NCBI project PRJNA562724 (https://www.ncbi.nlm.nih.gov/sra/?term=PRJNA562724) with accession number of 15 objects (SRR10031502, SRR10031501, SRR10031495, SRR10031494, SRR10031493, SRR10031492, SRR10031491, SRR10031490, SRR10031489, SRR10031488, SRR10031500, SRR10031499, SRR10031498, SRR10031497, SRR10031496). Any reasonable requests are available from the corresponding author.

## Abbreviations

BVDV: Bovine viral diarrhea virus
COG: Clusters of Orthologous Groups of proteins
CP: Cytopathogenic biotype
DEGs: Differentially expressed genes
DMEM: Dulbecco’s modified eagle medium
FBS: Fetal bovine serum
FC: Fold change
FPKM: The reads per kilobase of exon per million fragments mapped
GO: Gene ontology
IFA: Indirect immunofluorescence assay
IFN: Interferon
KEGG: Kyoto Encyclopedia of Genes and Genomes
MDBK: Madin-Darby bovine kidney cells
MOI: Multiplicity of infection
NCP: Non-cytopathogenic biotype
PAMP: Pathogen-associated molecular pattern
PBS: Phosphate buffer saline
PPI: Protein to protein interaction network
RT-qPCR: Reverse transcription real-time quantitative PCR
