# Supplementary material for: RNA-Seq based transcriptome analysis during bovine viral diarrhoea virus (BVDV) infection
Source: BMC Genomics. 2019 Oct 24;20:774. doi: 10.1186/s12864-019-6120-4 (PMC6813989; doi:10.1186/s12864-019-6120-4)
Supplement: Supplementary file 1 — Additional file 1: Table S1. Length distribution of transcripts. Table S2. The annotation of novel transcripts/genes compared with NR, Swiss-Prot, and Pfam databases. Table S3. Nodes centrality analysis of PPI network of group Mock vs. MBV2h. Table S4. Nodes centrality analysis of PPI network of group Mock vs. MBV24h. Table S5. Nodes centrality analysis of PPI network of group MBV2h vs. MBV6h. [file 12864_2019_6120_MOESM1_ESM.docx]

Table S1 Length distribution of transcripts.

| Length | Number |
| --- | --- |
| 0~200 | 3740 |
| 201~400 | 2094 |
| 401~600 | 2230 |
| 601~800 | 2545 |
| 801~1000 | 4015 |
| 1001~1200 | 2916 |
| 1201~1400 | 2769 |
| 1401~1600 | 2805 |
| 1601~1800 | 2631 |
| >1800 | 28184 |
| total | 53929 |

Table S2 The annotation of novel transcripts/genes compared with NR, Swiss-Prot, and Pfam databases.

| Database | New transcript number（percent） | New gene number（percent） |
| --- | --- | --- |
| NR | 25295(94.91%) | 1573(66.68%) |
| Swiss-Prot | 23570(88.44%) | 784(33.23%) |
| Pfam | 19341(72.57%) | 306(12.97%) |
| Total_anno | 25320(95.01%) | 1579(66.94%) |
| Total | 26651 | 2359 |

Table S3 Nodes centrality analysis of PPI network of group Mock vs. MBV2h

| Accession ID | Node ID | Node Name | Degree Centrality | Closeness Centrality | Betweenness Centrality |
| --- | --- | --- | --- | --- | --- |
| ENSBTAG00000039861 | 0 | OAS1Y | 0.329545455 | 0.523349938 | 0.329319151 |
| ENSBTAG00000004322 | 1 | FOS | 0.306818182 | 0.512812691 | 0.190227558 |
| ENSBTAG00000010069 | 2 | EGR1 | 0.215909091 | 0.477556818 | 0.096360744 |
| ENSBTAG00000023026 | 3 | PAI2 | 0.159090909 | 0.444238901 | 0.074925927 |
| ENSBTAG00000015582 | 4 | HMOX1 | 0.147727273 | 0.446836789 | 0.045342873 |
| ENSBTAG00000014835 | 5 | SPARC | 0.147727273 | 0.3859045 | 0.044359004 |
| ENSBTAG00000006367 | 6 | CTGF | 0.147727273 | 0.404280904 | 0.04067238 |
| ENSBTAG00000046158 | 7 | CFB | 0.125 | 0.374554367 | 0.030607558 |
| ENSBTAG00000020661 | 8 | ABCA1 | 0.113636364 | 0.358728126 | 0.033552658 |
| ENSBTAG00000019382 | 9 | PKDCC | 0.113636364 | 0.408604764 | 0.026937674 |
| ENSBTAG00000000706 | 10 | ADAMTS1 | 0.113636364 | 0.391841492 | 0.043602458 |
| ENSBTAG00000018237 | 11 | Bt.111268 | 0.113636364 | 0.40215311 | 0.038770644 |
| ENSBTAG00000008612 | 12 | C1R | 0.102272727 | 0.413022113 | 0.02919189 |
| ENSBTAG00000004840 | 13 | C1S | 0.102272727 | 0.353745791 | 0.012908332 |
| ENSBTAG00000007392 | 14 | SERPINA14 | 0.102272727 | 0.383965281 | 0.010484164 |
| ENSBTAG00000004063 | 15 | SERPINA5 | 0.102272727 | 0.372727273 | 0.011994846 |
| ENSBTAG00000013929 | 16 | RRAD | 0.102272727 | 0.41983017 | 0.020730023 |
| ENSBTAG00000045728 | 17 | SCD | 0.102272727 | 0.415266798 | 0.03469294 |
| ENSBTAG00000004104 | 18 | RUNX2 | 0.090909091 | 0.374554367 | 0.005964027 |
| ENSBTAG00000012519 | 19 | XDH | 0.090909091 | 0.370917917 | 0.03538596 |
| ENSBTAG00000008464 | 20 | ABCB9 | 0.090909091 | 0.374554367 | 0.033934914 |
| ENSBTAG00000010179 | 21 | COL5A3 | 0.079545455 | 0.367351399 | 0.019250022 |
| ENSBTAG00000005574 | 22 | CLU | 0.079545455 | 0.417536016 | 0.020007525 |
| ENSBTAG00000043553 | 23 | Bt.99682 | 0.068181818 | 0.406431335 | 0.023009114 |
| ENSBTAG00000005754 | 24 | PPM1K | 0.068181818 | 0.362128393 | 0.012378101 |
| ENSBTAG00000006039 | 25 | ARHGDIB | 0.068181818 | 0.370917917 | 0.007060916 |
| ENSBTAG00000002317 | 26 | PTN | 0.068181818 | 0.341112013 | 0.004906687 |
| ENSBTAG00000006599 | 27 | INHBE | 0.068181818 | 0.365593736 | 0.006263422 |
| ENSBTAG00000023411 | 28 | LOC525947 | 0.056818182 | 0.36042024 | 0.00068622 |
| ENSBTAG00000016819 | 29 | FABP3 | 0.056818182 | 0.326534577 | 0.007071049 |
| ENSBTAG00000011115 | 30 | CH25H | 0.056818182 | 0.36042024 | 0.002221281 |
| ENSBTAG00000017280 | 31 | C3 | 0.056818182 | 0.31705017 | 0.003372507 |
| ENSBTAG00000000745 | 32 | AQP1 | 0.056818182 | 0.362128393 | 0.02618511 |
| ENSBTAG00000007196 | 33 | TAGLN | 0.056818182 | 0.36042024 | 0.000378909 |
| ENSBTAG00000004399 | 34 | LRRN3 | 0.056818182 | 0.357051827 | 0.024705387 |
| ENSBTAG00000001785 | 35 | TGM3 | 0.056818182 | 0.355391121 | 0.005632889 |
| ENSBTAG00000020869 | 36 | DDC | 0.045454545 | 0.393861293 | 0.00423562 |
| ENSBTAG00000009812 | 37 | CXCL6 | 0.045454545 | 0.303210678 | 0.021377569 |
| ENSBTAG00000020056 | 38 | COL12A1 | 0.045454545 | 0.298473011 | 0.002430732 |
| ENSBTAG00000047957 | 39 | 100337286 | 0.045454545 | 0.353745791 | 0.00360348 |
| ENSBTAG00000022396 | 40 | SAA3 | 0.045454545 | 0.36042024 | 0.019852789 |
| ENSBTAG00000019368 | 41 | IGFBP7 | 0.045454545 | 0.306863819 | 0.003897213 |
| ENSBTAG00000001173 | 42 | PLXNA2 | 0.045454545 | 0.322401227 | 0.014137079 |
| ENSBTAG00000004150 | 43 | NRG1 | 0.045454545 | 0.363852814 | 0.038300171 |
| ENSBTAG00000009030 | 44 | NOX5 | 0.045454545 | 0.369126043 | 0.002981519 |
| ENSBTAG00000024449 | 45 | CENPF | 0.045454545 | 0.345742493 | 0.02361588 |
| ENSBTAG00000007062 | 46 | IGFBP5 | 0.045454545 | 0.3210466 | 0.008864146 |
| ENSBTAG00000014069 | 47 | PDK4 | 0.045454545 | 0.314440703 | 0.001198166 |
| ENSBTAG00000007101 | 48 | F3 | 0.045454545 | 0.362128393 | 0.000737981 |
| ENSBTAG00000008817 | 49 | LAMA4 | 0.034090909 | 0.284048665 | 0.001471832 |
| ENSBTAG00000006262 | 50 | LIMS2 | 0.034090909 | 0.309348546 | 0.004016013 |
| ENSBTAG00000007860 | 51 | ASPM | 0.034090909 | 0.342641663 | 0.010117182 |
| ENSBTAG00000002631 | 52 | SCNN1A | 0.034090909 | 0.284048665 | 0.00346968 |
| ENSBTAG00000002103 | 53 | - | 0.034090909 | 0.372727273 | 0.015518071 |
| ENSBTAG00000014103 | 54 | SH3GL2 | 0.034090909 | 0.271918473 | 0.0229989 |
| ENSBTAG00000001324 | 55 | SLCO2A1 | 0.034090909 | 0.336603925 | 0.000226402 |
| ENSBTAG00000033304 | 56 | C27H8orf4 | 0.034090909 | 0.288336192 | 0.00316284 |
| ENSBTAG00000018245 | 57 | SLC1A3 | 0.022727273 | 0.28617637 | 0.000705329 |
| ENSBTAG00000021570 | 58 | PLEKHG4 | 0.022727273 | 0.274852845 | 0.001023474 |
| ENSBTAG00000047962 | 59 | - | 0.022727273 | 0.342641663 | 0 |
| ENSBTAG00000021347 | 60 | CWH43 | 0.022727273 | 0.308101173 | 0.000318361 |
| ENSBTAG00000031579 | 61 | LOC532412 | 0.022727273 | 0.2538508 | 0 |
| ENSBTAG00000000191 | 62 | SLC25A20 | 0.022727273 | 0.34731405 | 0.000740122 |
| ENSBTAG00000017719 | 63 | AKAP6 | 0.022727273 | 0.280915775 | 0.00063857 |
| ENSBTAG00000009078 | 64 | MIA | 0.022727273 | 0.296159267 | 0 |
| ENSBTAG00000002266 | 65 | NPL | 0.022727273 | 0.273867709 | 0.007003608 |
| ENSBTAG00000004034 | 66 | SESN3 | 0.022727273 | 0.28617637 | 0.00039185 |
| ENSBTAG00000007275 | 67 | TREM2 | 0.022727273 | 0.281952365 | 0.00039185 |
| ENSBTAG00000008333 | 68 | ETV4 | 0.022727273 | 0.344185094 | 0 |
| ENSBTAG00000008858 | 69 | Bt.23349 | 0.022727273 | 0.341112013 | 0 |
| ENSBTAG00000007388 | 70 | ZC3H12D | 0.022727273 | 0.348899958 | 0.001319 |
| ENSBTAG00000008307 | 71 | PLCH1 | 0.022727273 | 0.341112013 | 0.00835825 |
| ENSBTAG00000016305 | 72 | ATP13A4 | 0.022727273 | 0.34731405 | 0 |
| ENSBTAG00000012052 | 73 | PADI4 | 0.022727273 | 0.290528863 | 0.000783699 |
| ENSBTAG00000019881 | 74 | ZNF474 | 0.011363636 | 0.011363636 | 0 |
| ENSBTAG00000003721 | 75 | CHST1 | 0.011363636 | 0.011363636 | 0 |
| ENSBTAG00000013982 | 76 | UACA | 0.011363636 | 0.259013867 | 0 |
| ENSBTAG00000004221 | 77 | ESM1 | 0.011363636 | 0.229456729 | 0 |
| ENSBTAG00000004337 | 78 | PDE1B | 0.011363636 | 0.011363636 | 0 |
| ENSBTAG00000004458 | 79 | MORN3 | 0.011363636 | 0.011363636 | 0 |
| ENSBTAG00000011896 | 80 | Bt.59467 | 0.011363636 | 0.281952365 | 0 |
| ENSBTAG00000001927 | 81 | ATP6V1C2 | 0.011363636 | 0.336603925 | 0 |
| ENSBTAG00000048249 | 82 | - | 0.011363636 | 0.273867709 | 0 |
| ENSBTAG00000005504 | 83 | LOC511340 | 0.011363636 | 0.302012217 | 0 |
| ENSBTAG00000013406 | 84 | CSRP2 | 0.011363636 | 0.211074837 | 0 |
| ENSBTAG00000017020 | 85 | S100G | 0.011363636 | 0.011363636 | 0 |
| ENSBTAG00000010798 | 86 | SLC23A1 | 0.011363636 | 0.266233766 | 0 |
| ENSBTAG00000022715 | 87 | DMBT1 | 0.011363636 | 0.011363636 | 0 |
| ENSBTAG00000037686 | 88 | SRPX2 | 0.011363636 | 0.276844532 | 0 |

Note: Degree Centrality: Degree Centrality is the most direct measure of node centrality in network analysis. The greater the degree of a node, the more important the node is in the network; Closeness Centrality: Closeness coefficient, the distance between the node and other nodes in the network, if very short, the point is the center of the whole network. The larger the value, the closer the node is to the center of the network; Betweeness Centrality: Median Centrality, which reflects the role of a node in connecting with other nodes. The larger the value, the more important the node is in maintaining the close connection of the whole network

Table S4 Nodes centrality analysis of PPI network of group Mock vs. MBV24h

| Accession ID | Node ID | Node Name | Degree Centrality | Closeness Centrality | Betweenness Centrality |
| --- | --- | --- | --- | --- | --- |
| ENSBTAG00000008545 | 0 | ATF3 | 0.119497 | 0.367758 | 0.22865 |
| ENSBTAG00000003835 | 1 | BMP4 | 0.100629 | 0.312594 | 0.086856 |
| ENSBTAG00000005339 | 2 | Bt.71502 | 0.09434 | 0.352219 | 0.138092 |
| ENSBTAG00000019716 | 3 | IL8 | 0.08805 | 0.31979 | 0.117533 |
| ENSBTAG00000043971 | 4 | LOC789485 | 0.08805 | 0.297709 | 0.042375 |
| ENSBTAG00000003711 | 5 | EPAS1 | 0.081761 | 0.325619 | 0.093528 |
| ENSBTAG00000008353 | 6 | CDKN1A | 0.075472 | 0.333434 | 0.050343 |
| ENSBTAG00000007689 | 7 | LPIN1 | 0.075472 | 0.276632 | 0.054665 |
| ENSBTAG00000017007 | 8 | TRIB3 | 0.075472 | 0.301296 | 0.084938 |
| ENSBTAG00000016254 | 9 | HDAC5 | 0.075472 | 0.314957 | 0.10561 |
| ENSBTAG00000007662 | 10 | grp78 | 0.069182 | 0.312594 | 0.088303 |
| ENSBTAG00000046409 | 11 | EGR2 | 0.069182 | 0.325619 | 0.054403 |
| ENSBTAG00000015133 | 12 | CCR7 | 0.062893 | 0.252601 | 0.015879 |
| ENSBTAG00000031544 | 13 | DDIT3 | 0.056604 | 0.311039 | 0.026509 |
| ENSBTAG00000000507 | 14 | NR4A1 | 0.056604 | 0.299133 | 0.027747 |
| ENSBTAG00000020355 | 15 | KLF4 | 0.056604 | 0.329047 | 0.053557 |
| ENSBTAG00000014496 | 16 | CCR6 | 0.050314 | 0.243738 | 0.003535 |
| ENSBTAG00000024552 | 17 | SHH | 0.050314 | 0.285474 | 0.01309 |
| ENSBTAG00000011131 | 18 | NMUR2 | 0.044025 | 0.243738 | 0.022132 |
| ENSBTAG00000009812 | 19 | CXCL6 | 0.044025 | 0.243264 | 0.000119 |
| ENSBTAG00000039292 | 20 | ADRA2B | 0.044025 | 0.243264 | 0.011146 |
| ENSBTAG00000014614 | 21 | ACTA2 | 0.044025 | 0.269478 | 0.041942 |
| ENSBTAG00000017024 | 22 | PPARGC1A | 0.044025 | 0.296298 | 0.044507 |
| ENSBTAG00000011274 | 23 | ENO2 | 0.044025 | 0.281617 | 0.029585 |
| ENSBTAG00000000163 | 24 | DDIT4 | 0.044025 | 0.292144 | 0.040022 |
| ENSBTAG00000018572 | 25 | FLT3 | 0.037736 | 0.274206 | 0.031015 |
| ENSBTAG00000021768 | 26 | CCNG2 | 0.037736 | 0.268898 | 0.019248 |
| ENSBTAG00000003222 | 27 | ASNS | 0.037736 | 0.286128 | 0.029851 |
| ENSBTAG00000003650 | 28 | NR4A2 | 0.037736 | 0.26491 | 0.002573 |
| ENSBTAG00000008182 | 29 | FOSB | 0.037736 | 0.284824 | 0.008624 |
| ENSBTAG00000007881 | 30 | IFIT1 | 0.037736 | 0.249079 | 0.010565 |
| ENSBTAG00000046885 | 31 | GPCR142 | 0.037736 | 0.242792 | 0 |
| ENSBTAG00000045728 | 32 | SCD | 0.037736 | 0.24092 | 0.013301 |
| ENSBTAG00000016896 | 33 | HERPUD1 | 0.031447 | 0.276021 | 0.022132 |
| ENSBTAG00000011105 | 34 | SLC38A2 | 0.031447 | 0.268898 | 0.055296 |
| ENSBTAG00000011839 | 35 | HMGCS1 | 0.031447 | 0.232412 | 0.007913 |
| ENSBTAG00000011982 | 36 | BATF3 | 0.031447 | 0.274808 | 0.008765 |
| ENSBTAG00000039861 | 37 | OAS1Y | 0.031447 | 0.248584 | 0.01462 |
| ENSBTAG00000016412 | 38 | CDKN2B | 0.031447 | 0.294206 | 0.012946 |
| ENSBTAG00000013108 | 39 | HK2 | 0.031447 | 0.297709 | 0.028064 |
| ENSBTAG00000015711 | 40 | BTG2 | 0.031447 | 0.288105 | 0.002426 |
| ENSBTAG00000001864 | 41 | NR4A3 | 0.031447 | 0.254659 | 0.001506 |
| ENSBTAG00000034075 | 42 | SLC7A11 | 0.031447 | 0.249576 | 0.020677 |
| ENSBTAG00000007763 | 43 | SLC1A4 | 0.031447 | 0.220525 | 0.034016 |
| ENSBTAG00000003994 | 44 | IGFBP3 | 0.031447 | 0.279726 | 0.0453 |
| ENSBTAG00000007196 | 45 | TAGLN | 0.031447 | 0.273008 | 0.024066 |
| ENSBTAG00000034918 | 46 | IFIT2 | 0.031447 | 0.241852 | 0.005953 |
| ENSBTAG00000003871 | 47 | CYP2B6 | 0.025157 | 0.236366 | 0.016579 |
| ENSBTAG00000013562 | 48 | SKP2 | 0.025157 | 0.265473 | 0.003962 |
| ENSBTAG00000013303 | 49 | ACSS2 | 0.025157 | 0.223282 | 0.001573 |
| ENSBTAG00000014335 | 50 | CYP17A1 | 0.025157 | 0.238622 | 0.009073 |
| ENSBTAG00000002363 | 51 | SESN2 | 0.025157 | 0.24711 | 0.011794 |
| ENSBTAG00000015347 | 52 | - | 0.025157 | 0.239536 | 0.000185 |
| ENSBTAG00000003707 | 53 | GRAP | 0.025157 | 0.25781 | 0.008592 |
| ENSBTAG00000015618 | 54 | GDF15 | 0.025157 | 0.289439 | 0.009761 |
| ENSBTAG00000016819 | 55 | FABP3 | 0.025157 | 0.235033 | 0.001137 |
| ENSBTAG00000037558 | 56 | GRO1 | 0.025157 | 0.240457 | 0 |
| ENSBTAG00000005754 | 57 | PPM1K | 0.025157 | 0.253626 | 0.025804 |
| ENSBTAG00000039035 | 58 | HSPA6 | 0.025157 | 0.226518 | 0.001825 |
| ENSBTAG00000012606 | 59 | ZNF541 | 0.025157 | 0.256751 | 0.011028 |
| ENSBTAG00000005741 | 60 | DLX2 | 0.025157 | 0.239996 | 0.011146 |
| ENSBTAG00000003532 | 61 | TLE4 | 0.025157 | 0.25108 | 0.004337 |
| ENSBTAG00000017691 | 62 | GPC5 | 0.025157 | 0.273606 | 0.001023 |
| ENSBTAG00000005947 | 63 | PLAU | 0.025157 | 0.274808 | 0.004916 |
| ENSBTAG00000047379 | 64 | CYP3A4 | 0.018868 | 0.203313 | 0.000842 |
| ENSBTAG00000006326 | 65 | ALDH1L2 | 0.018868 | 0.257279 | 0.007964 |
| ENSBTAG00000021570 | 66 | PLEKHG4 | 0.018868 | 0.237714 | 0.011776 |
| ENSBTAG00000011765 | 67 | GABARAPL1 | 0.018868 | 0.209093 | 0.014001 |
| ENSBTAG00000020512 | 68 | GJB1 | 0.018868 | 0.252601 | 0.011544 |
| ENSBTAG00000001592 | 69 | INSIG1 | 0.018868 | 0.216703 | 0.000178 |
| ENSBTAG00000007592 | 70 | RARG | 0.018868 | 0.224888 | 0 |
| ENSBTAG00000020869 | 71 | DDC | 0.018868 | 0.268898 | 0.022425 |
| ENSBTAG00000008333 | 72 | ETV4 | 0.018868 | 0.279102 | 0.006139 |
| ENSBTAG00000006287 | 73 | NEDD9 | 0.018868 | 0.255179 | 0.003917 |
| ENSBTAG00000046118 | 74 | SLC5A1 | 0.018868 | 0.177863 | 0.022212 |
| ENSBTAG00000006366 | 75 | Bt.101087 | 0.018868 | 0.263793 | 0.003177 |
| ENSBTAG00000033299 | 76 | IGFALS | 0.018868 | 0.214473 | 0.022212 |
| ENSBTAG00000008690 | 77 | glyT1 | 0.018868 | 0.21898 | 7.96E-05 |
| ENSBTAG00000021543 | 78 | MDFIC | 0.012579 | 0.234592 | 0.001609 |
| ENSBTAG00000019772 | 79 | OXTR | 0.012579 | 0.19207 | 0.011146 |
| ENSBTAG00000046556 | 80 | SOX4 | 0.012579 | 0.195067 | 0 |
| ENSBTAG00000038277 | 81 | HIST1H2BJ | 0.012579 | 0.233279 | 0 |
| ENSBTAG00000011278 | 82 | XYLB | 0.012579 | 0.224082 | 0.011146 |
| ENSBTAG00000040067 | 83 | HIPK4 | 0.012579 | 0.239078 | 0.002681 |
| ENSBTAG00000021307 | 84 | BNIP3L | 0.012579 | 0.199105 | 0.002032 |
| ENSBTAG00000004150 | 85 | NRG1 | 0.012579 | 0.198158 | 0.001125 |
| ENSBTAG00000017804 | 86 | BNIP3 | 0.012579 | 0.241386 | 0.010003 |
| ENSBTAG00000002998 | 87 | MFI2 | 0.012579 | 0.253626 | 0.001839 |
| ENSBTAG00000037778 | 88 | CXCL3 | 0.012579 | 0.19722 | 0 |
| ENSBTAG00000031886 | 89 | HIST1H2BL | 0.012579 | 0.233279 | 0 |
| ENSBTAG00000011237 | 90 | Bt.99599 | 0.012579 | 0.211212 | 0 |
| ENSBTAG00000016194 | 91 | FBXO32 | 0.012579 | 0.233715 | 0.001691 |
| ENSBTAG00000046467 | 92 | PTP4A3 | 0.012579 | 0.211212 | 0.000244 |
| ENSBTAG00000003329 | 93 | FST | 0.012579 | 0.232412 | 0.011146 |
| ENSBTAG00000010273 | 94 | EREG | 0.012579 | 0.188879 | 0.00062 |
| ENSBTAG00000011482 | 95 | SLC43A2 | 0.012579 | 0.208396 | 0 |
| ENSBTAG00000008197 | 96 | EPOR | 0.012579 | 0.212649 | 0.000322 |
| ENSBTAG00000008129 | 97 | CLSTN3 | 0.012579 | 0.234592 | 0.022132 |
| ENSBTAG00000013081 | 98 | PSPH | 0.012579 | 0.226108 | 0.011146 |
| ENSBTAG00000032068 | 99 | PLA2G4F | 0.012579 | 0.187182 | 0 |
| ENSBTAG00000010742 | 100 | PKD2L1 | 0.012579 | 0.211212 | 0 |
| ENSBTAG00000016276 | 101 | HAL | 0.012579 | 0.207359 | 0.011146 |
| ENSBTAG00000010371 | 102 | CHAC1 | 0.012579 | 0.268321 | 0 |
| ENSBTAG00000001294 | 103 | PPP1R15A | 0.012579 | 0.262684 | 0 |
| ENSBTAG00000016305 | 104 | ATP13A4 | 0.012579 | 0.186345 | 0.011146 |
| ENSBTAG00000003403 | 105 | PADI2 | 0.006289 | 0.006289 | 0 |
| ENSBTAG00000001444 | 106 | TNXB | 0.006289 | 0.179137 | 0 |
| ENSBTAG00000000745 | 107 | AQP1 | 0.006289 | 0.148325 | 0 |
| ENSBTAG00000010179 | 108 | COL5A3 | 0.006289 | 0.231551 | 0 |
| ENSBTAG00000000770 | 109 | PGM2L1 | 0.006289 | 0.214106 | 0 |
| ENSBTAG00000005260 | 110 | SPP1 | 0.006289 | 0.231551 | 0 |
| ENSBTAG00000047794 | 111 | DNAH1 | 0.006289 | 0.207016 | 0 |
| ENSBTAG00000007392 | 112 | SERPINA14 | 0.006289 | 0.158076 | 0 |
| ENSBTAG00000007388 | 113 | ZC3H12D | 0.006289 | 0.231551 | 0 |
| ENSBTAG00000006599 | 114 | INHBE | 0.006289 | 0.184421 | 0 |
| ENSBTAG00000005828 | 115 | MERTK | 0.006289 | 0.197532 | 0 |
| ENSBTAG00000005586 | 116 | GATM | 0.006289 | 0.216703 | 0 |
| ENSBTAG00000005504 | 117 | LOC511340 | 0.006289 | 0.169428 | 0 |
| ENSBTAG00000005304 | 118 | SRM | 0.006289 | 0.006289 | 0 |
| ENSBTAG00000001950 | 119 | RDH11 | 0.006289 | 0.006289 | 0 |
| ENSBTAG00000005063 | 120 | THEM6 | 0.006289 | 0.006289 | 0 |
| ENSBTAG00000003326 | 121 | NAIP | 0.006289 | 0.006289 | 0 |
| ENSBTAG00000004178 | 122 | ACOX2 | 0.006289 | 0.222487 | 0 |
| ENSBTAG00000004063 | 123 | SERPINA5 | 0.006289 | 0.231551 | 0 |
| ENSBTAG00000004034 | 124 | SESN3 | 0.006289 | 0.225293 | 0 |
| ENSBTAG00000003721 | 125 | CHST1 | 0.006289 | 0.191189 | 0 |
| ENSBTAG00000004010 | 126 | PAPPA | 0.006289 | 0.172943 | 0 |
| ENSBTAG00000004221 | 127 | ESM1 | 0.006289 | 0.252601 | 0 |
| ENSBTAG00000018160 | 128 | LAMA1 | 0.006289 | 0.006289 | 0 |
| ENSBTAG00000011187 | 129 | FAM13A | 0.006289 | 0.197532 | 0 |
| ENSBTAG00000015177 | 130 | PRSS23 | 0.006289 | 0.006289 | 0 |
| ENSBTAG00000017719 | 131 | AKAP6 | 0.006289 | 0.238622 | 0 |
| ENSBTAG00000019421 | 132 | DACT1 | 0.006289 | 0.006289 | 0 |
| ENSBTAG00000019665 | 133 | IL1RN | 0.006289 | 0.235476 | 0 |
| ENSBTAG00000017450 | 134 | KLHL24 | 0.006289 | 0.220137 | 0 |
| ENSBTAG00000019754 | 135 | PRKCDBP | 0.006289 | 0.006289 | 0 |
| ENSBTAG00000013861 | 136 | SLC8A1 | 0.006289 | 0.154177 | 0 |
| ENSBTAG00000016567 | 137 | HBQ1 | 0.006289 | 0.189739 | 0 |
| ENSBTAG00000020616 | 138 | DGKD | 0.006289 | 0.006289 | 0 |
| ENSBTAG00000020809 | 139 | SLC36A2 | 0.006289 | 0.206674 | 0 |
| ENSBTAG00000016275 | 140 | AMDHD1 | 0.006289 | 0.168288 | 0 |
| ENSBTAG00000015381 | 141 | ARHGAP18 | 0.006289 | 0.238622 | 0 |
| ENSBTAG00000017949 | 142 | LOC617396 | 0.006289 | 0.006289 | 0 |
| ENSBTAG00000015307 | 143 | Bt.110891 | 0.006289 | 0.006289 | 0 |
| ENSBTAG00000013953 | 144 | CALD1 | 0.006289 | 0.209093 | 0 |
| ENSBTAG00000018497 | 145 | SDPR | 0.006289 | 0.006289 | 0 |
| ENSBTAG00000024493 | 146 | DHRS3 | 0.006289 | 0.006289 | 0 |
| ENSBTAG00000013929 | 147 | RRAD | 0.006289 | 0.172943 | 0 |
| ENSBTAG00000024595 | 148 | HECA | 0.006289 | 0.230697 | 0 |
| ENSBTAG00000014053 | 149 | MAK | 0.006289 | 0.206674 | 0 |
| ENSBTAG00000024928 | 150 | TNFRSF1B | 0.006289 | 0.006289 | 0 |
| ENSBTAG00000011838 | 151 | PCED1B | 0.006289 | 0.006289 | 0 |
| ENSBTAG00000012128 | 152 | AASS | 0.006289 | 0.006289 | 0 |
| ENSBTAG00000031785 | 153 | HIST2H2BE | 0.006289 | 0.232845 | 0 |
| ENSBTAG00000038415 | 154 | SLC6A12 | 0.006289 | 0.148325 | 0 |
| ENSBTAG00000012052 | 155 | PADI4 | 0.006289 | 0.193557 | 0 |
| ENSBTAG00000008313 | 156 | KLF15 | 0.006289 | 0.211212 | 0 |
| ENSBTAG00000011624 | 157 | FGF21 | 0.006289 | 0.189165 | 0 |
| ENSBTAG00000011327 | 158 | OLFML3 | 0.006289 | 0.006289 | 0 |
| ENSBTAG00000031814 | 159 | SDS | 0.006289 | 0.18043 | 0 |

Note: Degree Centrality: Degree Centrality is the most direct measure of node centrality in network analysis. The greater the degree of a node, the more important the node is in the network; Closeness Centrality: Closeness coefficient, the distance between the node and other nodes in the network, if very short, the point is the center of the whole network. The larger the value, the closer the node is to the center of the network; Betweeness Centrality: Median Centrality, which reflects the role of a node in connecting with other nodes. The larger the value, the more important the node is in maintaining the close connection of the whole network

Table S5 Nodes centrality analysis of PPI network of group MBV2h vs. MBV6h

| Accession ID | Node ID | Node Name | Degree Centrality | Closeness Centrality | Betweenness Centrality |
| --- | --- | --- | --- | --- | --- |
| ENSBTAG00000016740 | 0 | ACLY | 0.846154 | 0.866667 | 0.279915 |
| ENSBTAG00000011839 | 1 | HMGCS1 | 0.769231 | 0.8125 | 0.130769 |
| ENSBTAG00000012432 | 2 | FDFT1 | 0.769231 | 0.8125 | 0.220085 |
| ENSBTAG00000007840 | 3 | HMGCR | 0.615385 | 0.722222 | 0.009829 |
| ENSBTAG00000013303 | 4 | ACSS2 | 0.615385 | 0.722222 | 0.009829 |
| ENSBTAG00000001592 | 5 | INSIG1 | 0.538462 | 0.684211 | 0.002137 |
| ENSBTAG00000007689 | 6 | LPIN1 | 0.538462 | 0.684211 | 0.041453 |
| ENSBTAG00000012059 | 7 | MVD | 0.461538 | 0.65 | 0 |
| ENSBTAG00000017690 | 8 | Bt.24269 | 0.384615 | 0.619048 | 0 |
| ENSBTAG00000002129 | 9 | KLF5 | 0.230769 | 0.52 | 0.004274 |
| ENSBTAG00000010909 | 10 | SLC30A1 | 0.230769 | 0.541667 | 0.006838 |
| ENSBTAG00000002765 | 11 | CYP24A1 | 0.153846 | 0.5 | 0 |
| ENSBTAG00000021013 | 12 | TUBB4A | 0.076923 | 0.464286 | 0 |
| ENSBTAG00000046708 | 13 | PDP2 | 0.076923 | 0.481481 | 0 |

Note: Degree Centrality: Degree Centrality is the most direct measure of node centrality in network analysis. The greater the degree of a node, the more important the node is in the network; Closeness Centrality: Closeness coefficient, the distance between the node and other nodes in the network, if very short, the point is the center of the whole network. The larger the value, the closer the node is to the center of the network; Betweeness Centrality: Median Centrality, which reflects the role of a node in connecting with other nodes. The larger the value, the more important the node is in maintaining the close connection of the whole network.
