# Supplementary material for: RNA-Seq based transcriptome analysis during bovine viral diarrhoea virus (BVDV) infection
Source: BMC Genomics. 2019 Oct 24;20:774. doi: 10.1186/s12864-019-6120-4 (PMC6813989; doi:10.1186/s12864-019-6120-4)
Supplement: Supplementary file 2 — Additional file 2: Figure S1. Venn analysis of functional annotation and classification within GO, COG and KEGG database. Figure S2. Functional annotation of the genes obtained from RNA-Seq. The different colours of the pie chart represent different GO terms, and the area represents the relative proportion of genes/transcripts in the GO Term. The number of genes were shown behind the subsets. Go terms of level 2 were annotated within the three Go categories, biological progress (BP), cellular progress (CC), and molecular function (MF). The functional annotation was performed using Blast2go. Figure S3. Function classification in Clusters of Orthologous Groups of Proteins (COG) of genes from RNA-Seq. Capital letters on x axis indicated the COG categories as listed on the right of histogram; y axis indicated the number of gene. Figure S4. Pathway annotation of genes from RNA-Seq. KEGG Pathway of level 2 within the seven categories of KEGG pathway as listed on the right of histogram were annotated. x axis indicated the number of genes annotated to the pathway; y axis indicated the name of the KEGG pathway. Figure S5. Venn analysis of differential expression genes from group Mock vs. MBV2h, Mock vs. MBV6h, Mock vs. MBV12h, Mock vs. MBV24h. Figure S6. Dynamic changes of BVDV RNA in MDBK cells infected with 10 MOI BVDV. a: BVDV positive strand RNA; b: BVDV negative strand RNA. Copies of BVDV positive or negative strand RNA was quantified by strand specific SYBR Green real-time PCR and shown as copies of positive or negative strand RNA of BVDV per microgram total RNA of cells. Figure S7. GO enrichment analysis of DEGs in comparison groups MBV2h vs. MBV6h(a), MBV6h vs. MBV 12 h(b), MBV12h vs. MBV24h(c). GO terms are on the x axis. Enrichment ratio of genes shown as GO terms for BP, CC, and MF. * means GO terms with significant enrichment. Figure S8. KEGG Pathway enrichment analysis of DEGs in comparison groups MBV2h vs. MBV6h (a), MBV6h vs. MBV12h (b), MBV12h vs. MBV24h (c). [file 12864_2019_6120_MOESM2_ESM.docx]

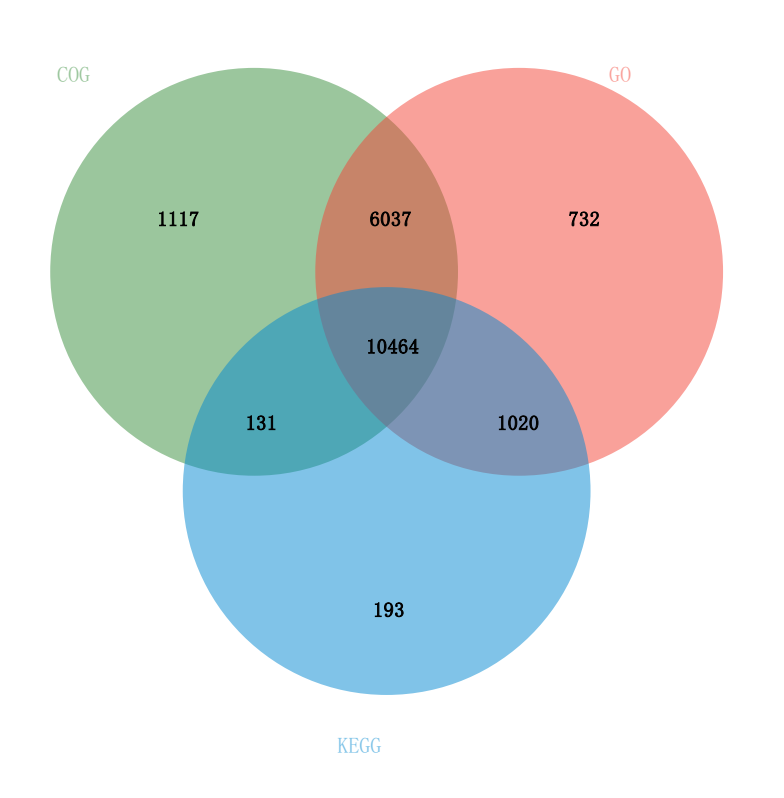


**Fig. S1** Venn analysis of functional annotation and classification within GO, COG and KEGG database.


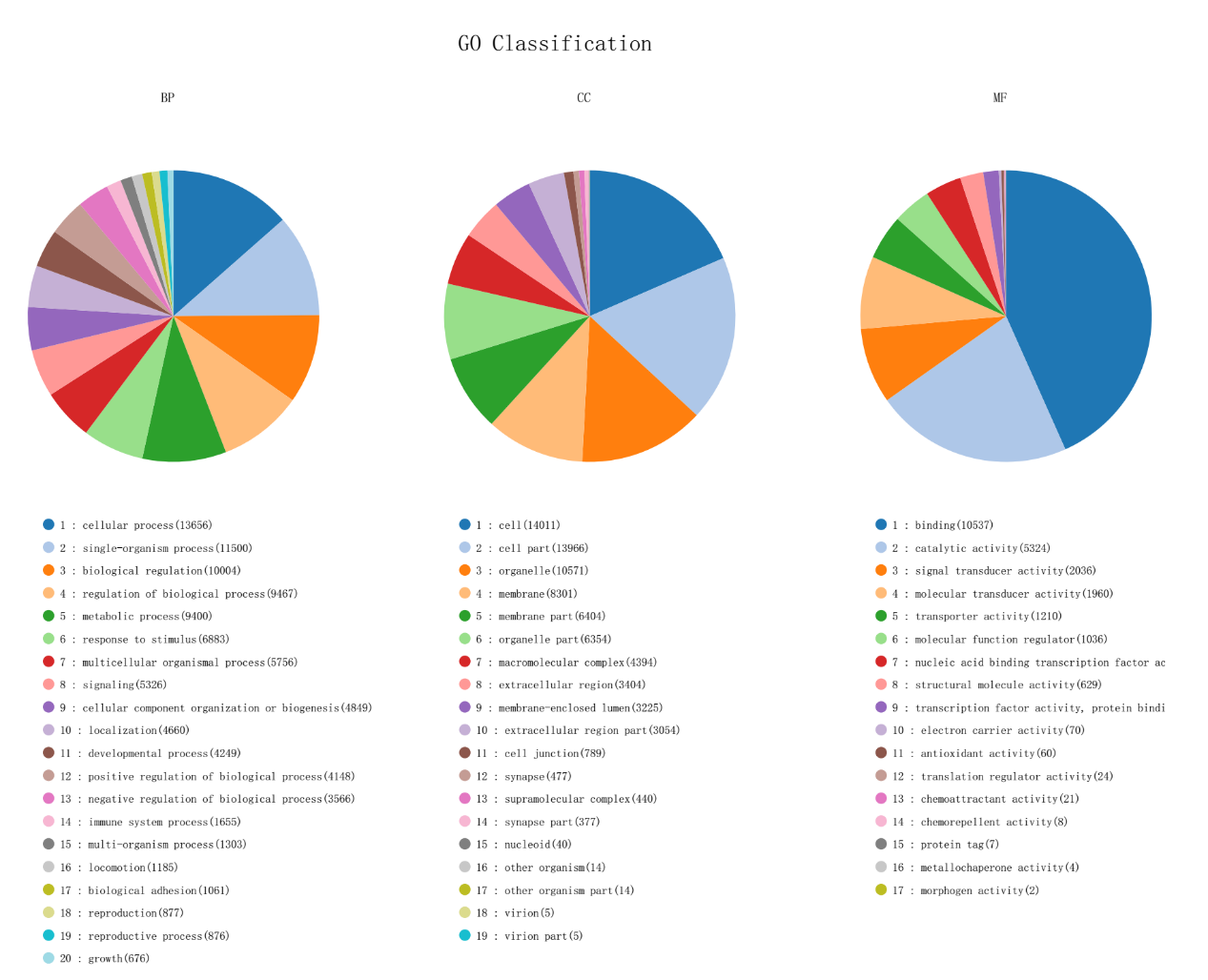


Fig. S2 Functional annotation of the genes obtained from RNA-Seq. The different colors of the pie chart represent different GO terms, and the area represents the relative proportion of genes/transcripts in the GO Term. The number of genes were shown behind the subsets. Go terms of level 2 were annotated within the three Go categories, biological progress (BP), cellular progress (CC), and molecular function (MF). The functional annotation was performed using Blast2go.


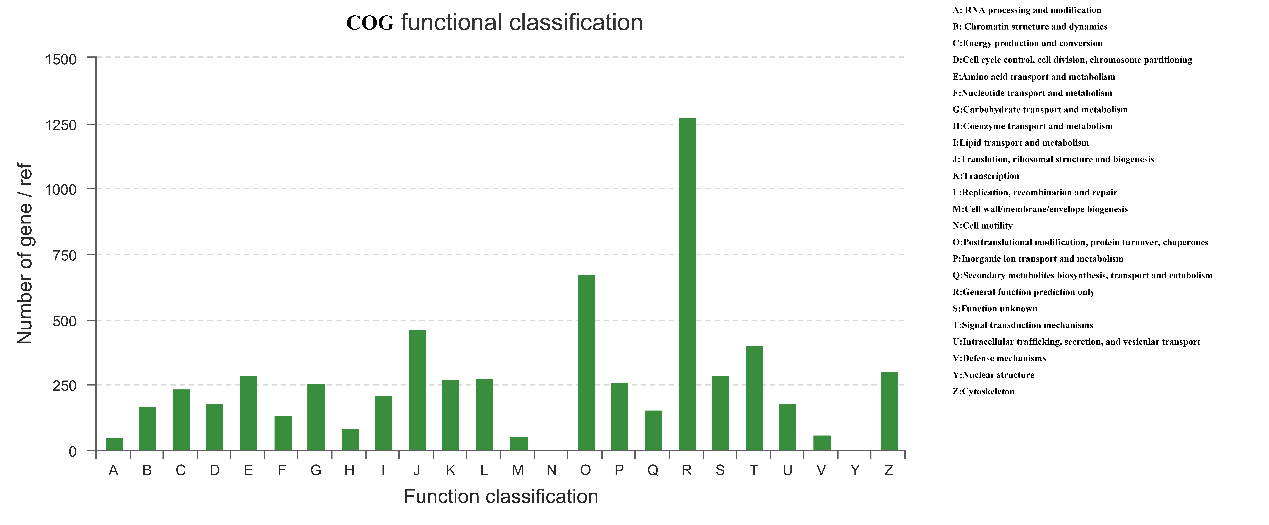


**Fig. S3** Function classification in Clusters of Orthologous Groups of Proteins (COG) of genes from RNA-Seq. Capital letters on x axis indicated the COG categories as listed on the right of histogram; y axis indicated the number of gene.


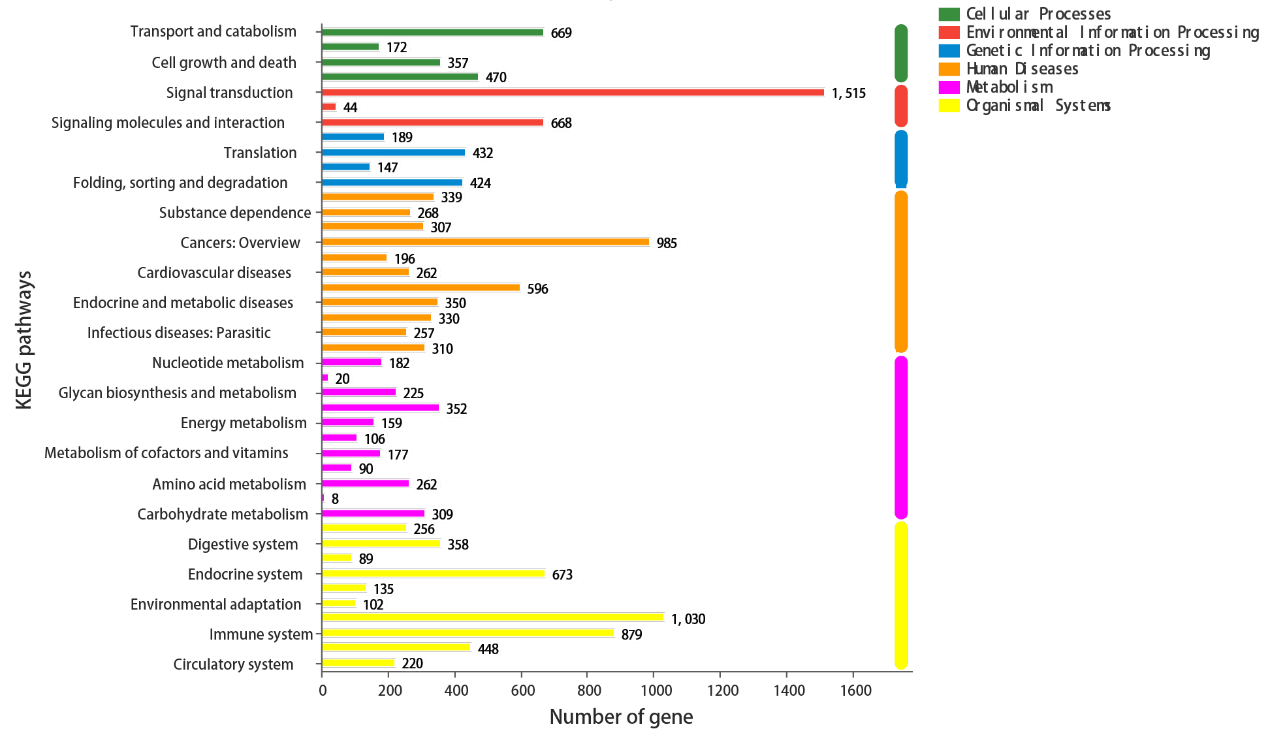


**Fig. S****4** Pathway annotation of genes from RNA-Seq. KEGG Pathway of level 2 within the seven categories of KEGG pathway as listed on the right of histogram were annotated. x axis indicated the number of genes annotated to the pathway; y axis indicated the name of the KEGG pathway.


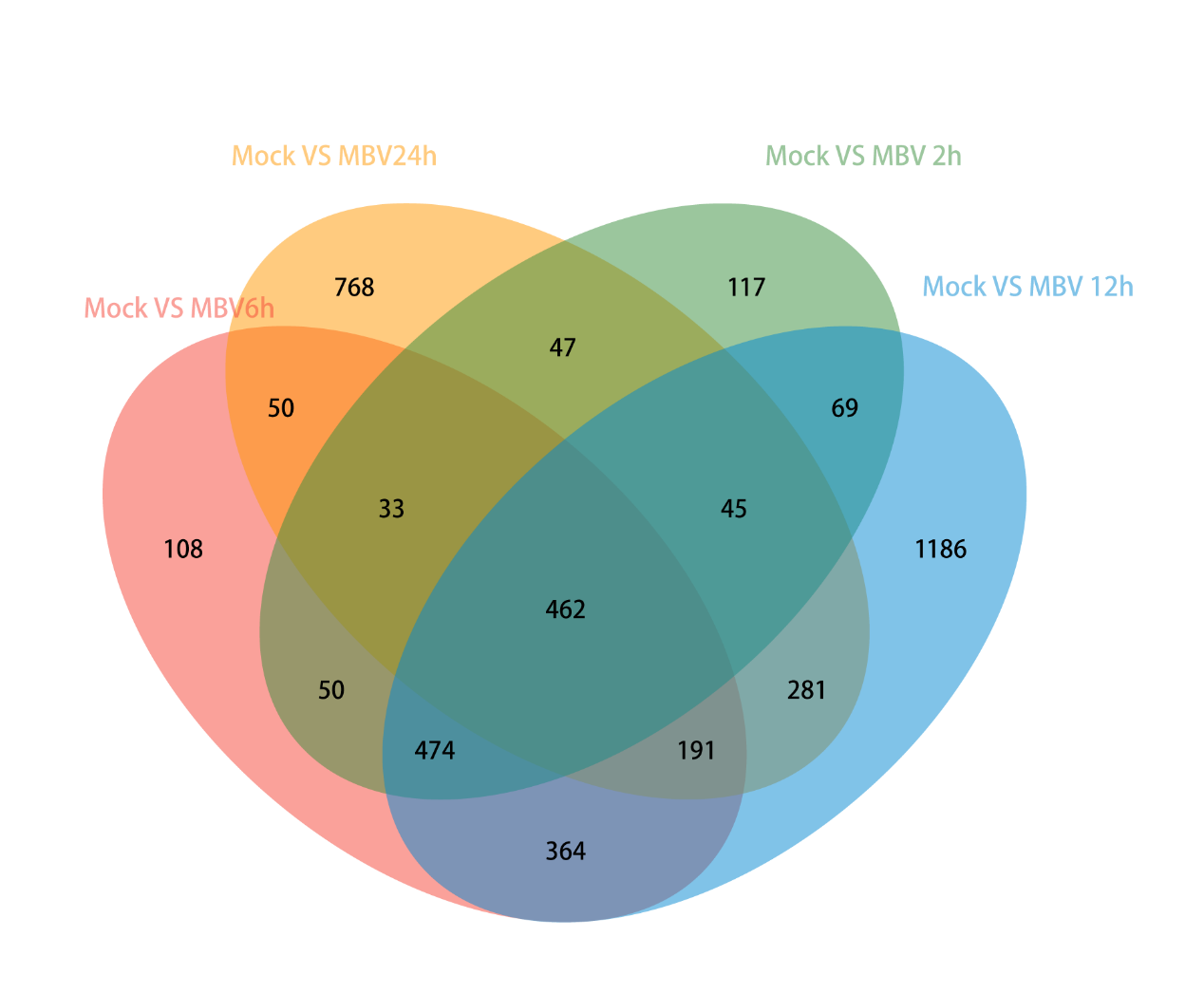


**Fig. S5** Venn analysis of differential expression genes from group Mock vs. MBV2h, Mock vs. MBV6h, Mock vs. MBV12h, Mock vs. MBV24h.





**Fig. S6** Dynamic changes of BVDV RNA in MDBK cells infected with 10 MOI BVDV.

a: BVDV positive strand RNA; b: BVDV negative strand RNA. Copies of BVDV positive or negative strand RNA was quantified by strand specific SYBR Green real-time PCR and shown as copies of positive or negative strand RNA of BVDV per microgram total RNA of cells.


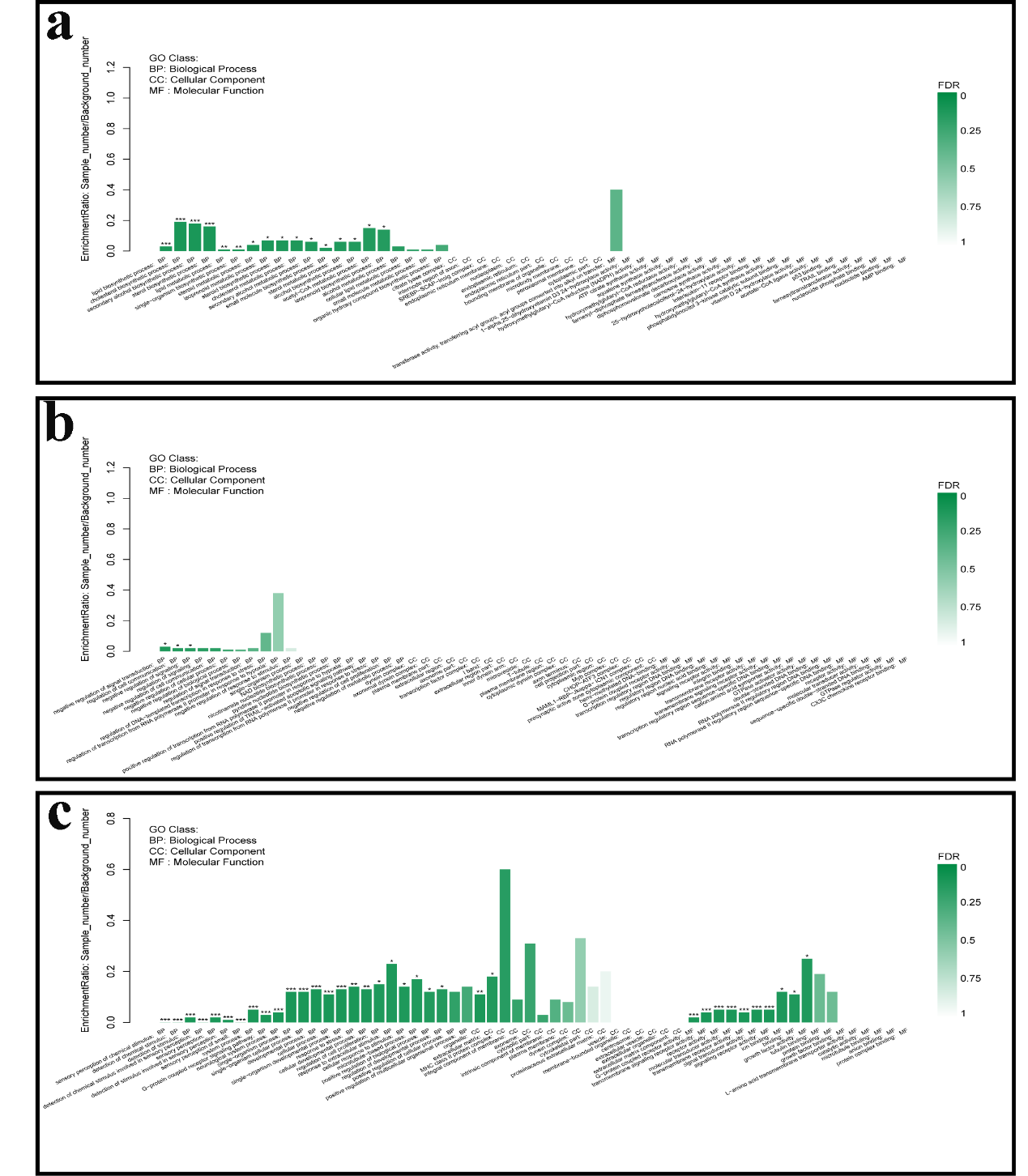


**Fig. S7** GO enrichment analysis of DEGs in comparison groups MBV2h vs. MBV 6h(a), MBV6h vs. MBV 12h(b), MBV12h vs. MBV 24h(c). GO terms are on the x axis. Enrichment ratio of genes shown as GO terms for BP, CC, and MF. * means GO terms with significant enrichment


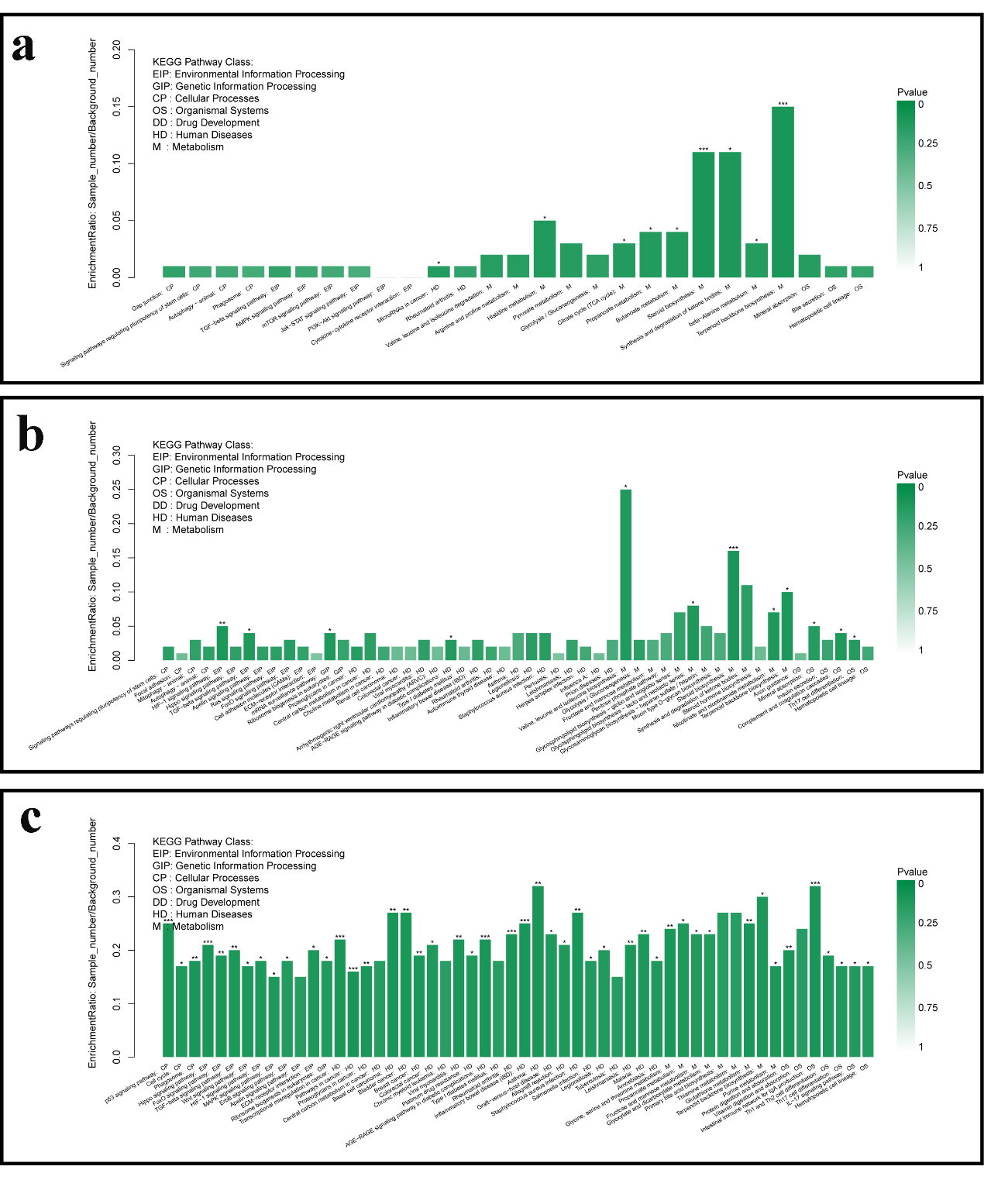


**Fig.S8** KEGG Pathway enrichment analysis of DEGs in comparison groups MBV2h vs. MBV 6h (a), MBV6h vs. MBV 12h (b), MBV12h vs. MBV 24h (c). The name of KEGG pathway are on the x axis. Enrichment ratio of genes shown as the name of KEGG pathway for seven categories. * means KEGG Pathway with significant enrichment.


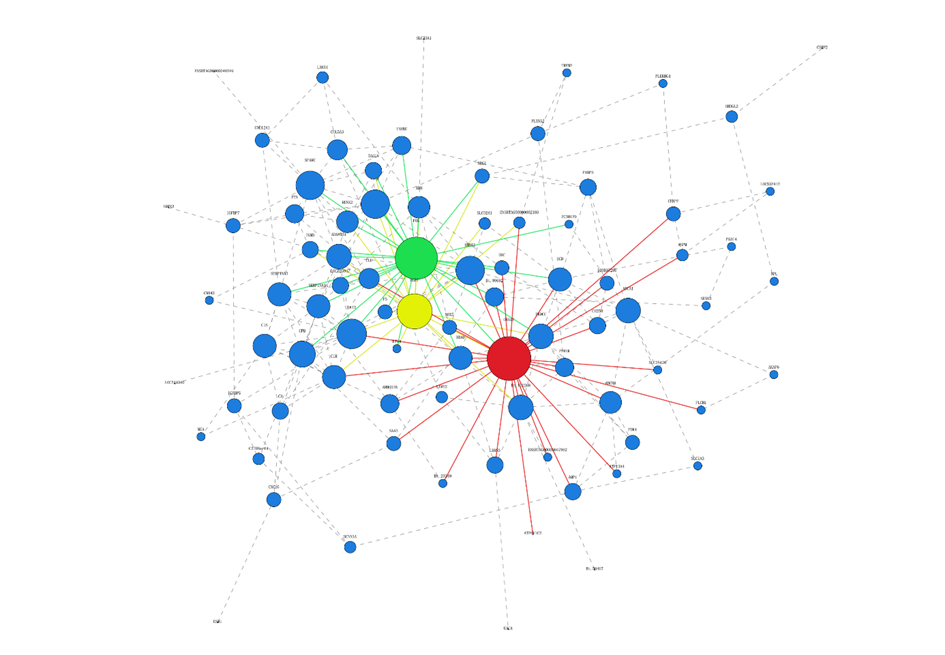


Fig. S9 Protein–Protein Interaction Network Analysis of DEGs with FDR≤0.05 and |Log2FC|≥2 in comparison group Mock vs. MBV2h. OAS1Y, FOS and EGR1 were three genes with the most node degree in the analysis, labelled in red blot, green blot and yellow blot, respectively.


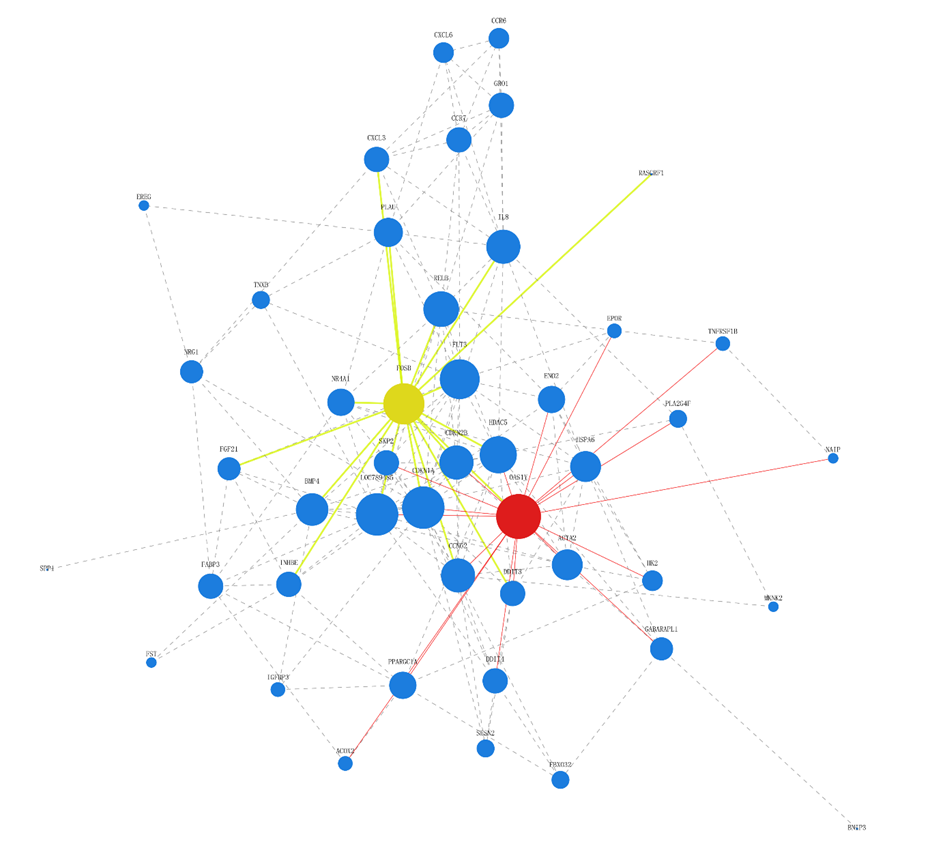


**Fig. S10** Protein–Protein Interaction Network Analysis of DEGs with FDR≤0.05 and |Log2FC|≥2 in comparison group Mock vs. MBV24h. OAS1Y and FOSB were the two genes with the most node degree, labeled in red blot and yellow blot, respectively.
